# Targeted activity of the small molecule kinase inhibitor Pz-1 towards RET and TRK kinases

**DOI:** 10.1038/s41598-021-95612-4

**Published:** 2021-08-09

**Authors:** Marialuisa Moccia, Donglin Yang, Naga Rajiv Lakkaniga, Brendan Frett, Nicholas McConnell, Lingtian Zhang, Annalisa Brescia, Giorgia Federico, Lingzhi Zhang, Paolo Salerno, Massimo Santoro, Hong-yu Li, Francesca Carlomagno

**Affiliations:** 1 Dipartimento di Medicina Molecolare e Biotecnologie Mediche, Università Di Napoli “Federico II”, Via S. Pansini 5, 80131 Naples, Italy; 2 Department of Pharmaceutical Sciences, College of Pharmacy, University of Arkansas for Medical Sciences, Little Rock, AR 72205 USA; 3 Synactix Pharmaceuticals, Inc., Tucson, AZ 85718 USA; 4 Istituto di Endocrinologia ed Oncologia Sperimentale del CNR, 80131 Naples, Italy

**Keywords:** Cancer therapy, Drug development, Targeted therapies, Cancer, Drug discovery

## Abstract

We have recently described Pz-1, a benzimidazole-based type-2 RET and VEGFR2 inhibitor. Based on a kinome scan, here we show that Pz-1 is also a potent (IC_50_ < 1 nM) TRKA/B/C inhibitor. Pz-1 potently inhibited proliferation of human cancer cells carrying either RET- or TRKA oncoproteins (IC_50_ ~ 1 nM), with a negligible effect against RET- and TRKA-negative cells. By testing mutations, known to mediate resistance to other compounds, RET G810R/S, but not L730I/V, E732K, V738A and Y806N, showed some degree of resistance to Pz-1. In the case of TRKA, G595R and F589L, but not G667C, showed some degree of resistance. In xenograft models, orally administered Pz-1 almost completely inhibited RET- and TRKA-mutant tumours at 1–3 mg/kg/day but showed a reduced effect on RET/TRKA-negative cancer models. The activity, albeit reduced, on RET/TRKA-negative tumours may be justified by VEGFR2 inhibition. Tumours induced by NIH3T3 cells transfected by RET G810R and TRKA G595R featured resistance to Pz-1, demonstrating that RET or TRKA inhibition is critical for its anti-tumourigenic effect. In conclusion, Pz-1 represents a new powerful kinase inhibitor with distinct activity towards cancers induced by oncogenic RET and TRKA variants, including some mutants displaying resistance to other drugs.

## Introduction

Several tyrosine kinase inhibitors (TKIs) have been approved for cancer treatment, since the initial approval of Imatinib in 2001^1,2^. RET and TRKs (TRKA, TRKB and TRKC) receptor tyrosine kinases (RTKs) represent relevant drug targets because of their involvement in several cancer types^3,4^.

TRK proteins are generally activated via intra- or inter-chromosomal rearrangements in which the kinase domain is fused to partner polypeptides that contain dimerization motifs, such as coiled-coil, zinc finger or WD domains^5,6^. Constitutive dimerization mediates activation of the kinase followed by transphosphorylation and signalling. A variety of adult and paediatric cancers display TRKs fusions. Some specific cancers, such as secretory breast carcinoma, MASC (mammary analogue secretory carcinoma), congenital mesoblastic nephroma, and infantile fibrosarcoma, are positive for TRKs fusions in more than 90% of cases^7–9^. Others, like papillary thyroid cancer (PTC), Spitzoid neoplasm, gastrointestinal stromal tumour and paediatric glioma, display TRKs rearrangements in around 5–25% of cases^10,11^. In addition, a vast group of neoplasms, including breast, lung (non-small cell lung cancer -NSCLC) and colorectal (CRC) carcinoma, melanoma and leukaemia are positive for TRKs fusions in less than 5% of cases^4,5,10^. Finally, TRKs overexpression (breast, lung, skin carcinoma)^11^, splice variants and in-frame deletions (AML and neuroblastoma)^12,13^ have been reported.

RET is commonly activated by gene fusions in different cancers. In these cases, partner genes provide dimerization motives that promote constitutive kinase activation^3,14–17^. PTC, both in adults and children, is the neoplasm with the highest prevalence of RET fusions (5–30%), especially when associated to ionizing radiations^18^. Less frequently (< 1%) RET kinase fusions are found in NSCLC, CRC, breast and salivary duct carcinoma^19–23^, chronic myeloproliferative disorders^24,25^ and Spitzoid neoplasm^26^. Beside gene fusions, medullary thyroid carcinoma (MTC) (60–70%) displays oncogenic point mutations in RET^17^. In 25% of cases the mutations are germinal and associated to Multiple Endocrine Neoplasia type 2 (MEN2)^27,28^. In addition to MTC, MEN2 patients display frequently pheocromocytoma and, depending on the specific subtype, parathyroid hyperplasia (MEN2A) or neuroma/ganglioneuroma of the intestinal tract (MEN2B)^27,28^. Overexpression of RET has been reported in some tumours including breast and pancreatic adenocarcinoma^3,16^.

In this light, RET and TRKs represent appealing targets for cancer therapy. In 2018, the first TRK inhibitor, larotrectinib, was approved for treatment of adult and paediatric TRK fusion-positive cancer^29^. Subsequently, two other TRK TKIs, entrectinib and selitrectinib, have been evaluated in clinical trials and entrectinib has been approved for solid tumours that display TRK rearrangements^30–33^. Vandetanib and cabozantinib, with inhibitory activity against RET, VEGFR2 and other targets, have been approved for their ability to improve progression-free survival of MTC patients^34,35^ and tested in other RET-driven cancers^36,37^. Novel potent and VEGFR-sparing TKIs (pralsetinib and selpercatinib) have been identified and demonstrated substantial clinical efficacy in RET-driven cancers; both have been approved as RET-targeted cancer drugs^38–43^.

Mutations in the TRKA kinase, in the solvent-exposed region (G595R), at the gatekeeper residue (F589L) or in the activation loop "DFG" motif (G667C), confer resistance to various TRKA inhibitors^44,45^. F589L, G595R and G667C have been described in colorectal cancer patients who were initially responsive to entrectinib or larotrectinib and then developed secondary resistance to treatment^44,45^.

RET mutations affecting the solvent-exposed region (G810A), the gatekeeper residue (V804L/M), or the hinge region (Y806C) mediate resistance to vandetanib and cabozantinib^46–49^. Moreover, Liu et al. identified several additional RET mutations, including L730I/V (in the roof of the solvent front), E732K (in the glycine-rich loop), V738A (in the β2 strand), Y806N, G810S, A807V, V871I, M918T and F998V that confer resistance to one or more RET inhibitors^50^. Pralsetinib and selpercatinib proved to inhibit V804L/M RET mutants^38^ but not different mutants at G810 (G810R/S/C) or Y806 (Y806C/N) residues^51,52^. E732K and V738A also featured resistance to both pralsetinib and selpercatinib, while L730I/V was resistant to pralsetinib only^52,53^. An additional resistance forming mutation was identified in a NSCLC patient who developed resistance to vandetanib as a consequence of the S904F mutation in the CCDC6-RET activation loop^54^.

We have recently reported the identification of a novel TKI, named Pz-1, able to potently inhibit RET and VEGFR2 kinases (IC_50_ < 1 nM)^55^. Here we show that Pz-1 is a potent inhibitor of TRK kinases as well, that is active against several RET and TRKA mutants that are resistant to other TKIs and that is effective in xenografts of human MTC, lung and colon carcinoma cells driven by RET or TRKA oncogenes.

## Materials and methods

The study was carried out in compliance with the ARRIVE guidelines.

### Compound

Pz-1 was synthesized as described in Frett et al^55^. For in vitro experiments, Pz-1 was dissolved in DMSO at a concentration of 50 mM and stored at -80 °C. Final dosing solution was freshly prepared for each experiment from the stock solution; the equivalent amount of vehicle (DMSO) was used as control. For in vivo experiments, Pz-1 was dissolved at a concentration of 1 mg/ml in 20% Tween20, 80% water and 0.1% Xanthan gum. This formulation was stored at 4 °C.

### Molecular modelling

RET DFG-out homology model was built using SWISS-MODEL (https://swissmodel.expasy.org) from the primary sequence of RET obtained from Uniprot and VEGFR-2 DFG-out crystal structure (PDB# 2OH4) as the template. The protein was prepared using Protein Preparation Wizard module of Maestro 2018–2 software suite. Bond orders were properly adjusted and missing hydrogens were added. Using Gromacs 5.1, energy minimization was performed using default parameters to obtain the optimum structure. For molecular docking, a grid was defined around the ATP binding site covering the hinge region, DFG residues and the residues of the αC-helix facing the ATP pocket. Using Autodock Vina, the ligand was docked into the grid. The results were visualized using Maestro 2018-2 suite^55^.

The crystal structure of TRKA (4GT5) was retrieved from Protein Data Bank. The protein was prepared to add the missing hydrogens, adjust the bond orders and bond angles. The grid was defined around the co-crystallized ligand. The results were analyzed using the academic version of Maestro software. Using Autodock Vina, the ligand was docked into the grid. The results were visualized using Maestro 2018–2 suite.

### TRK binding assay

TRK (A, B, C) kinases T7 phage strains were prepared in an E. coli host derived from the BL21 strain. E. coli were grown to log-phase and infected with T7 phage and incubated with shaking at 32 °C until lysis. The lysates were centrifuged and filtered to remove cell debris. Streptavidin-coated magnetic beads were treated with biotinylated small molecule ligands for 30 min at room temperature to generate affinity resins for kinase assays. The liganded beads were blocked with excess biotin and washed with SeaBlock blocking buffer containing 1% BSA, 0.05% Tween 20, 1 mM DTT to remove unbound ligand and to reduce non-specific binding (Thermo Fisher Scientific, Watham, MA). Binding reactions were assembled by combining kinases, liganded affinity beads, and test compounds in 1 × binding buffer (20% SeaBlock, 0.17 × PBS, 0.05% Tween 20, 6 mM DTT). Test compounds were prepared as 111X stocks in 100% DMSO. K_D_s were determined using an 11-point threefold compound dilution series with three DMSO control points. The TRK kinase binding assays were outsourced to DiscoveRx, Fremont Ca.

### Cell cultures

Human HEK 293 T cells were from American Type Culture Collection (ATCC, Manassas, VA, USA) and were grown in DMEM supplemented with 10% fetal bovine serum (GIBCO, Thermo Fisher Scientific, Waltham, MA, USA). Transient transfections of the different TRKA and RET mutants (all coding the short RET-9 isoform) vectors were carried out with the Fugene reagent according to manufacturer's instructions (Promega, Milano, Italy). Ba/F3 murine pro-B cells stably expressing NCOA4-RET, RET/C634R and RET/M918T mutant proteins (all expressing in the long RET-51 isoform) or TPM3-TRKA were generated by electroporation. Parental and transformed Ba/F3 cells were cultured in RPMI supplemented with 10% fetal bovine serum (FBS) (Life Technologies), supplemented with 10 ng/ml IL-3 in the case of parental cells (Peprotech, Cranbury, New Jersey, USA). Mouse NIH3T3 fibroblasts stably transfected with NCOA4-RET, NCOA4-RET G810R, TPM3-TRKA or TPM3-TRKA G595R were generated by electroporation and were grown in DMEM supplemented with 5% calf serum (CS) (GIBCO). Human medullary thyroid carcinoma (MTC) TT cells were from ATCC and grown in RPMI 1640 (Life Technologies, Carlsbad, CA, USA) supplemented with 20% FBS; human MTC MZ-CRC-1 cells were kindly provided by R.F. Gagel (MD Anderson, Houston, TX, USA) and grown in DMEM (Life Technologies) supplemented with 10% FBS. Human papillary thyroid cancer (PTC) cell lines TPC-1, kindly provided by M. Nagao (National Cancer Center Research Institute, Tokyo, Japan), and B-CPAP were grown in DMEM with 10% FBS. Human anaplastic thyroid cancer (ATC) 8505C cells (European Collection of Cell Cultures – ECACC, Salisbury, UK), were grown in DMEM supplemented with 10% FBS. SV40-immortalized human thyroid follicular epithelial Nthy-ori-3-1 cells were obtained from ECACC and were grown in DMEM with 10% FBS. Human colorectal carcinoma (CRC) cell lines KM12 and HCT-116, kindly provided by A. Bardelli (Candiolo Cancer Institute, Torino, Italy) and D. Grieco (Dipartimento di Medicina Molecolare e Biotecnologie Mediche, Naples, Italy) respectively, were grown in RPMI with 10% FBS. Human non-small cell lung cancer (NSCLC) cell lines Lc-2/ad, A549, Calu-1 and PC-9 were obtained from ECACC. Lc-2/ad cells were grown in RPMI 1640/HAM’S F12 (1:1) (Lonza, Walkersville, MD USA), A549 were grown in DMEM, Calu-1 were grown in EMEM (Lonza) and PC-9 were grown in RPMI 1640, all supplemented with 10% FBS. All cell culture media were supplemented with 2 mM L-glutamine and 100 units/ml penicillin–streptomycin (Life Technologies, Carlsbad, CA, USA). Authentication of all cell lines, by DNA fingerprint, was routinely performed at BMR Genomics (Aviano, Italy).

### Site-directed mutagenesis

Mutagenesis of pBABE-puro NCOA4-RET and TPM3-TRKA vectors were performed using QuikChange II XL Site-Directed Mutagenesis Kit according to manufacturer's protocol (Agilent Technologies, Santa Clara, CA, USA). Primers are listed in Supplementary information, Table S2, and were designed using QuikChange Primer Design Program available online.

### Growth curves and cell viability assay

Nthy-ori-3-1, 8505C, TPC-1, and HCT-116 (5000/well), B-CPAP, A549, Calu-1, PC-9, KM12, and NCOA4-RET and TPM3-TRKA-transformed NIH3T3 cells (10,000/well), MZ-CRC-1 and Lc-2/ad (100,000/well), and TT and Ba/F3 (200,000/well) were seeded in 6-well plates. TPC-1, Nthy-ori-3-1 and TT cells were grown in 2%, 5% and 20% FBS respectively. NIH3T3 transfectants were grown in 2.5% CS. All the other cell lines were kept in 10% FBS. The day after plating, cells were counted and various concentrations of drug or vehicle were added to the medium and changed every 2–3 days. Cells were counted in triplicate every 2–3 days (or daily for 4 consecutive days in the case of Ba/F3) and the number of cells of the last day of the experiment was used to calculate growth inhibition. Growth inhibition by the NCI-60 Human Tumour Cell Lines Screen was outsourced to National Cancer Institute, Division of Cancer Treatment and Diagnosis (DCTD)^56^. Cell viability was measured using the CellTiter 96 AQueous One Solution Cell Proliferation Assay according to manufacturer's instructions (MTS; Promega, Madison, WI, USA). Briefly, TT (50,000/well), MZ-CRC-1 (50,000/well), TPC-1 (10,000/well), Lc-2/ad (10,000/well) and KM12 (30,000/well) were seeded in 96-well plates; the day after plating, different concentrations of Pz-1 were added to the medium and incubated for 48 (TPC-1, Lc-2/ad, KM12) or 72 h (TT, MZ-CRC-1). At the end of treatment, CellTiter 96 reagent was added to each well and then incubated at 37 °C for 7 (TPC-1), 60 (MZ-CRC-1 and KM12), 100 (Lc-2/ad) or 165 (TT) minutes. The absorbance at 490 nm was analyzed using a microplate reader (Synergy H1; Bio Tek, Winooski, VT, USA).

### Protein studies

Immunoblotting experiments were performed as previously described^55^. Anti-RET is a polyclonal antibody raised against the tyrosine kinase protein fragment of human RET. Anti-pY1062 and anti-pY905 are phospho-specific affinity-purified RET polyclonal antibodies^55^. Antibodies for phospho-VEGFR2/KDR (pY1175) (#2478), VEGFR2/KDR (#2479), phospho-PLCγ1 (Tyr783) (#2821), PLCγ1 (#2822), phospho-TRKA (Tyr490) (#9141), phospho-TRKA (Tyr674/675) (#4621), TRK (pan) (A7H6R) (#92,991), phospho-p44/42 MAPK (Erk1/2) (Thr202/Tyr204) (#4370) and p44/42 MAPK (Erk1/2) (#4695) were from Cell Signaling Technology (Danvers, MA, USA). Anti-SHC (H-108) (#sc-1695) was from Santa Cruz Biotechnology (Santa Cruz, CA, USA). Anti phospho-tyrosine, clone 4G10 (05–321) and anti-phospho-SHC (Y317) (#07–206) were from Merck Millipore (Billerica, MA, USA). Secondary antibodies coupled to horseradish peroxidase were purchased from Bio-Rad. Densitometric analysis was performed using ImageJ software.

### Cell cycle analysis

For flow cytometry analysis, TT and MZ-CRC-1 (1 × 10^6^/dish), Nthy-ori-3-1, Lc-2/ad, A549, Calu-1 and PC-9 (5 × 10^5^/dish), TPC-1, B-CPAP, 8505C, KM12 and HCT-116 (2 × 10^5^/dish) were plated in 60 mm dishes in complete medium; the next day cells were treated with different concentrations of Pz-1 or vehicle. After 48 h, cells were harvested by trypsinization and fixed in cold 70% ethanol for 16 h at -20° C. After washing with PBS, cells were treated with RNAse A (100 U/ml), stained with propidium iodide (25 μg/ml) (Sigma-Aldrich, Saint Louis, MO, USA) for 20 min and analyzed with a BD Accuri C6 Flow Cytometer (BD Biosciences, San Jose, CA, USA) interfaced with the Cell Quest software (BD Biosciences). A minimum of 3 × 10^4^ events were acquired for each experiment.

### Mouse experiments

Animal studies were conducted at the Dipartimento di Medicina Molecolare e Biotecnologie Mediche in accordance with Italian regulations for experimentation on animals; The study was approved by the Italian Ministry of health (Authorization n. 1023/2015-PR). Female SCID (for TT, MZ-CRC-1 and 8505C cells) or BALB/c nu/nu (for KM12, HCT-116 and transformed NIH3T3 cells) mice were purchased from Charles River Laboratories (Wilmington, MA, USA). All manipulations were performed while the animals were under isoflurane gas anesthesia. No mouse showed signs of wasting or other signs of toxicity. Animals were treated for different times due to the different doubling times displayed by the cell lines used.

TT (15 × 10^6^/mouse), MZ-CRC-1 and 8505C cells (10 × 10^6^/mouse) were inoculated subcutaneously into dorsal portions (both sides) of mice. Once palpable tumours were measured, mice were sorted into treatment groups to achieve equal distribution of tumour size in all groups, as described hereafter. As far as TT cells, after 5 weeks, tumour xenografts (average volume ranging ~ 70–110 mm^3^) were evident on both sides in 28 mice, and only in one side in the remaining 6 mice. Tumour-bearing animals were randomly assigned to receive Pz-1 (0.3, 1.0 or 3.0 mg/kg, daily) (26 mice, 48 tumours) or vehicle (8 mice, 14 tumours) by oral gavage for 28 consecutive days. As far as MZ-CRC-1 cells, after 19 days, tumour xenografts (average volume ~ 50 mm^3^) were evident on both sides in 26 mice and in only one side in 7 mice (2 mice showed no tumour). Tumour-bearing animals were randomly assigned to receive Pz-1 (0.3, 1.0 or 3.0 mg/kg) (35 mice, 61 tumours) or vehicle (9 mice, 14 tumours) by oral gavage for 28 consecutive days. As far as 8505C cells, after 2 weeks, tumour xenografts (average volume ~ 50 mm^3^) were evident on both sides in 16 mice and in only one side in 5 mice (3 mice showed no tumour). Animals were randomly assigned to receive Pz-1 (0.3, 1.0, or 3.0 mg/kg) (18 mice, 28 tumours) or vehicle (6 mice, 9 tumours) by oral gavage for 25 consecutive days daily.

KM12 and HCT-116 cells (10 × 10^6^/mouse) were inoculated subcutaneously into dorsal portion (both sides) of mice (24 mice for each cell line). After 5 days, when tumours were just palpable, animals were randomly assigned to receive Pz-1 or vehicle control. KM12 tumour-bearing animals were treated with vehicle (8 mice), 3.0 mg/kg Pz-1 (8 mice), or 0.6 mg/kg Pz-1 (7 mice) by oral gavage for 16 consecutive days. HCT-116 tumour-bearing animals (8 mice/group) were treated with vehicle, 3.0 mg/kg, or 0.6 mg/kg Pz-1 by oral gavage for 37 consecutive days.

For transformed NIH3T3 xenografts, mice were inoculated subcutaneously into both flanks with 2 × 10^5^ NIH3T3 NCOA4-RET, NIH3T3 NCOA4-RET G810R, NIH3T3 TPM3-TRKA and NIH3T3 TPM3-TRKA G595R cells. After 4 days, before tumour became visible, animals were randomly assigned to receive Pz-1 or vehicle control. NIH3T3 NCOA4-RET and NIH3T3 NCOA4-RET G810R tumour-bearing animals were treated with vehicle (6mice for NIH3T3 NCOA4-RET and 7 mice for NIH3T3 NCOA4-RET G810R), 0.3, 1 or 3 mg/kg (6 mice/group) Pz-1 by oral gavage for 16 consecutive days. NIH3T3 TPM3-TRKA and NIH3T3 TPM3-TRKA G595R tumour-bearing animals were treated with vehicle (6 mice/group), 0.3 mg/Kg (6 mice/group), 1 mg/Kg (5 mice for NIH3T3 TPM3-TRKA and 6 mice for NIH3T3 TPM3-TRKA G595R) or 3 mg/kg (6 mice/group) Pz-1 by oral gavage for 17 consecutive days.

Tumour volumes (V) were calculated by the rotational ellipsoid formula: V = A × B^2^/2 (A = axial diameter; B = rotational diameter). Tumour diameters were measured with calipers every 3–4 days for TT, MZ-CRC-1, 8505C, KM12 and NIH3T3 transfectants and every 7 days for HCT-116 xenografts. Three hours after the last dose, mice were euthanized by cervical dislocation; tumours were excised and snap-frozen in liquid nitrogen. In MZ-CRC-1 xenografts, for measurement of RET phosphorylation and Pz-1 plasma concentration, tumours and blood were collected 2, 4, 8, and 24 h following the last dose. Plasma was separated by centrifugation and stored frozen (− 80 °C) until analysis. Drug concentration determination was outsourced to Pharmaron (Rushden, UK) and performed by LC–MS/MS.

For single time point in vivo target inhibition, mice (SCID or BALB/c nu/nu, as reported above) were injected with TT, MZ-CRC-1, 8505C, KM12, HCT-116, NIH3T3 NCOA4-RET, NIH3T3 NCOA4-RET G810R, NIH3T3 TPM3-TRKA and NIH3T3 TPM3-TRKA G595R cells (6–8 mice per cell line). When tumours reached a volume of 400–500 mm^3^, mice were subjected to treatment with Pz-1 or vehicle (2 doses in 24 h) and then tumours were collected and snap frozen in liquid nitrogen. For time course in vivo target inhibition, BALB/c nu/nu mice (26 mice per cell line) were injected with NIH3T3 NCOA4-RET and NIH3T3 TPM3-TRKA cells (2 × 10^5^). When tumours reached a volume of 400–500 mm^3^, mice (2 mice/group) were treated with a single oral dose of Pz-1 (0.3, 1 or 3 mg/Kg) or vehicle; tumours were collected after 2, 8, 12 and 24 h and snap frozen in liquid nitrogen.

Frozen tumours were homogenized in lysis buffer by using the Mixer Mill MM300 (Qiagen, Germantown, MD, USA). Samples were clarified twice by centrifugation at 10,000 × *g*. Tumour proteins phosphorylation were assayed according to standard Western blot procedures.

### Statistical analysis

The statistical significance of the results was evaluated as previously reported^55^. Briefly, the unpaired Student’s *t* test by the Instat software program (Graphpad Software Inc, San Diego, CA, USA) was performed to compare cell growth. *P* values were two-sided, and differences were statistically significant at *P* < 0.02. Kruskal–Wallis, Mann–Whitney, and Dunn’s Multiple Comparison tests (InStat program) were used to compare tumour growth. IC_50_ doses for cell growth were calculated through a curve fitting analysis from last day of growth curves using the PRIZM software (Graphpad Software Inc). *P* values were statistically significant at < 0.05.

## Results

### Pz-1 inhibits TRK and RET kinases

At a kinome scan (at 50 nM) performed against a panel of 91 kinases from each kinome cluster, Pz-1, previously characterized as a potent RET inhibitor, was incidentally found to be also able to bind to TRK kinases^55^. We performed an in vitro binding assay using recombinant TRKA, TRKB and TRKC kinases. As shown in Table 1, similar to RET kinase (< 1 nM)^55^, Pz-1 was able to bind the three TRKs with a K_D_ < 1 nM. Accordingly, Pz-1 was able to inhibit phosphorylation of TRKA-derived (TPM3-TRKA) as well as RET-derived (CCDC6-RET, NCOA4-RET) chimeric oncoproteins transiently expressed in HEK 293 T cells at one-digit nanomolar concentration (Supplementary information, Figure S1).

**Table 1:** Pz-1 binding to TRK kinases.

Kinase	K_D_ (nM)
TRKA	0.34
TRKB	0.78
TRKC	0.31

K_D_ of Pz-1 for the indicated kinases.

**Figure 1:**
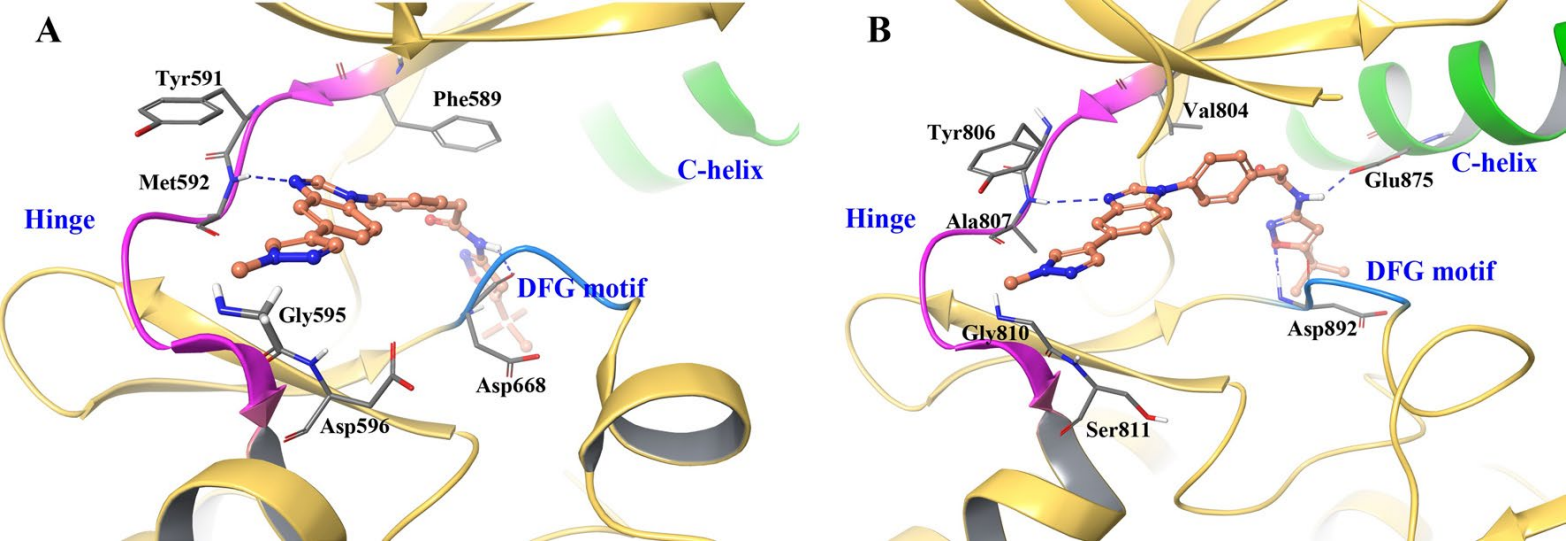
Proposed binding pose and interactions of Pz-1 in TRKA (**A**) and RET (**B**). Kinase structure: yellow ribbons; hinge residues: pink ribbon; DFG residues: blue ribbon; αC-helix: green ribbon; carbon atoms: orange; nitrogen atoms: blue; oxygen atoms: red.

We performed computational studies to compare the Pz-1 binding mode to RET and TRKA (Fig. 1). In the RET kinase, residues 805–811 form the ‘hinge’ region, while residues 892–894 represent the DFG motif. Molecular docking of Pz-1 in RET shows two hydrogen bonds, with A807 and D892, which stabilize the inhibitor in the ATP-binding pocket. V804, the ‘gatekeeper residue’ of RET is not involved in strong interactions with Pz-1, being located 5 Å away from the inhibitor, consistent with the notion that mutations at V804 did not influence the sensitivity to Pz-1^55^. Y806, a hinge residue involved in resistance to several TKIs, did not interact with the drug^47,50,52^. In contrast, G810, at the solvent front, laid very close to the drug. The binding pose of Pz-1 in TRKA was highly similar to that in RET, supporting the notion that Pz-1 acted as a TRK inhibitor as well. Noteworthy, G595, that corresponds to RET G810, mapped very close to the predicted drug binding site.

Stable expression of chimeric TPM3-TRKA and CCDC6-RET as well as MTC-associated point-mutant RET C634R and RET M918T oncoproteins promoted IL-3-independent growth of Ba/F3. This effect was blunted by Pz-1 treatment (IC_50_ of 0.3–1.6 nM) (Supplementary information, Figure S2). Accordingly, Pz-1 inhibited RET and TRKA phosphorylation (Supplementary information, Figure S3). No effect was observed on IL-3-driven growth of parental Ba/F3 up to 100 nM Pz-1 concentration (Supplementary information, Figure S2). In the same cell model, cell proliferation IC_50_ was: i) 9.7 or 16.5 nM with pralsetinib^39,53^, 341 nM with cabozantinib^39^, 793 nM with vandetanib^39^, and 8.0 nM with selpercatinib^53^ in the case of KIF5B-RET cells; ii) 8.0 nM with larotrectinib in the case of MPRIP-TRKA cells^57^ and 2.8 nM with entrectinib in the case of TEL-TRKA^58^.

### Pz-1 inhibits phosphorylation and signalling in RET and TRKA mutant cancer cells

**Figure 2:**
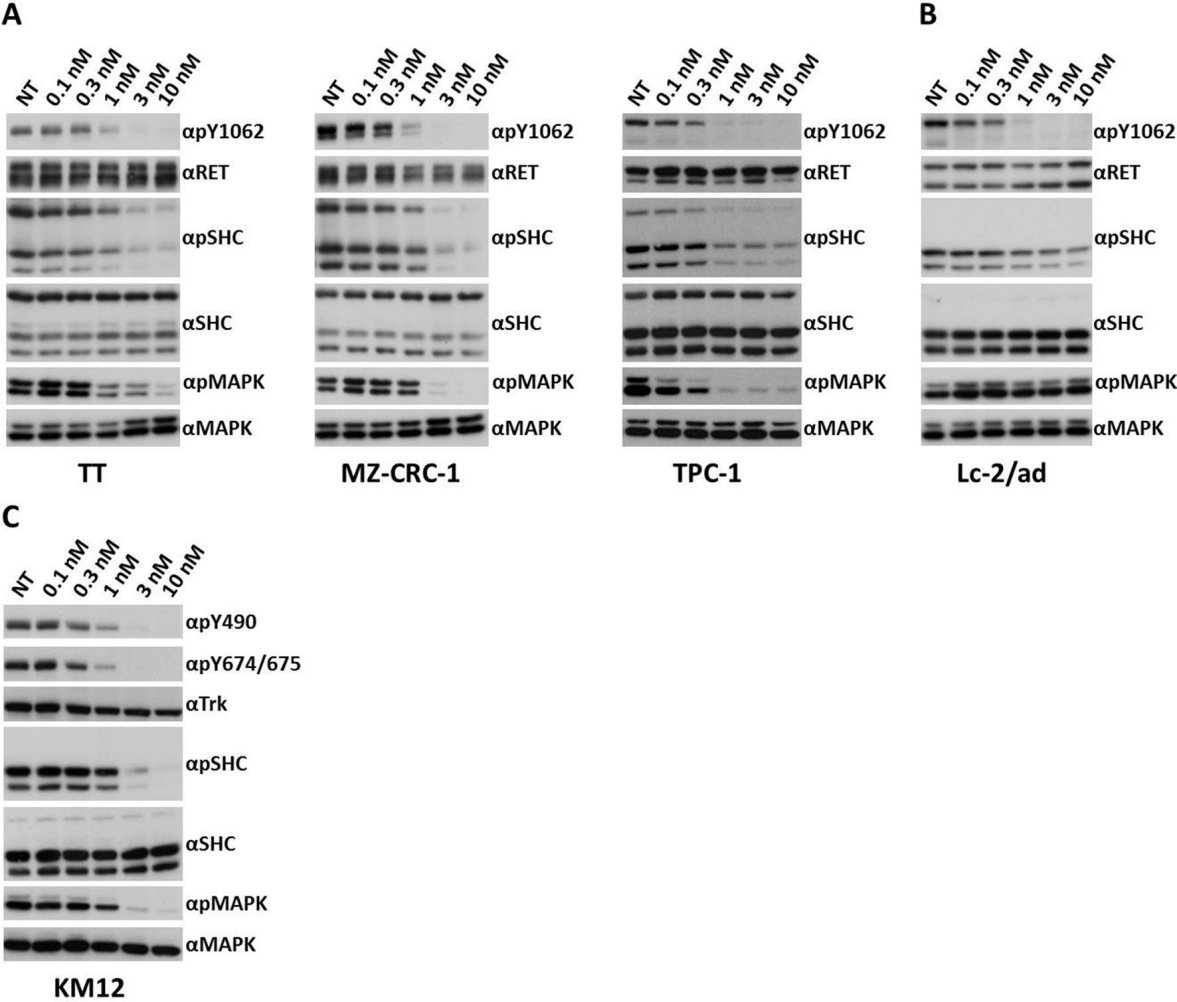
Serum-starved RET-positive thyroid cancer cell lines TT, MZ-CRC-1, TPC-1 (**A**), RET-positive lung cancer cell line Lc-2/ad (**B**) and TRKA-positive colon cancer cell line KM12 (**C**) were treated for 2 h with the indicated concentrations of Pz-1. Total cell lysates (50 μg) were subjected to immunoblotting with the indicated antibodies.

TT and MZ-CRC-1 (MTC cells harbouring RET C634W and M918T mutants, respectively), TPC-1 (PTC cells harbouring the CCDC6-RET fusion), Lc-2/ad (NSCLC cells harbouring the CCDC6-RET fusion) and KM12 (CRC cells harbouring the TPM3-TRKA fusion) were selected to explore the ability of Pz-1 to inhibit RET- and TRKA-derived oncoproteins in human cancer cell lines endogenously harbouring oncogenic versions of the two RTKs. RET autophosphorylation was strongly inhibited by 1 nM Pz-1 in MTC, PTC, and NSCLC cells. Consistently, at the same dose, RET-dependent SHC and ERK1/2 (MAPK) phosphorylation was also inhibited (with the exception of Lc-2/ad cells in which ERK1/2 inhibition was not detectable) (Fig. 2A,B). Similarly, TRKA autophosphorylation and SHC and ERK1/2 phosphorylation were inhibited by 1 nM Pz-1 in KM12 cells (Fig. 2C). As a control, human cancer cell lines of the same tissues but harbouring oncogenes other than RET or TRKA were used. In particular, we selected thyroid cancer-derived cell lines (B-CPAP and 8505C) harbouring BRAF V600E, the SV40-immortalized thyrocyte cell line (Nthy-ori-3-1), NSCLC cell lines (A549, PC-9 and Calu-1) harbouring KRAS G12S, HER1 LREA del and KRAS G12C, respectively, and the CRC cell line (HCT-116) harbouring KRAS G38A. No inhibition of SHC and ERK1/2 phosphorylation was observed in any of these cell lines up to 100 nM Pz-1 with the exception of Nthy-ori-3-1 cells in which ERK phosphorylation was partially inhibited at this concentration (Supplementary information, Figure S4).

### Pz-1 selectively inhibits proliferation of RET and TRKA driven cancer cells

**Figure 3:**
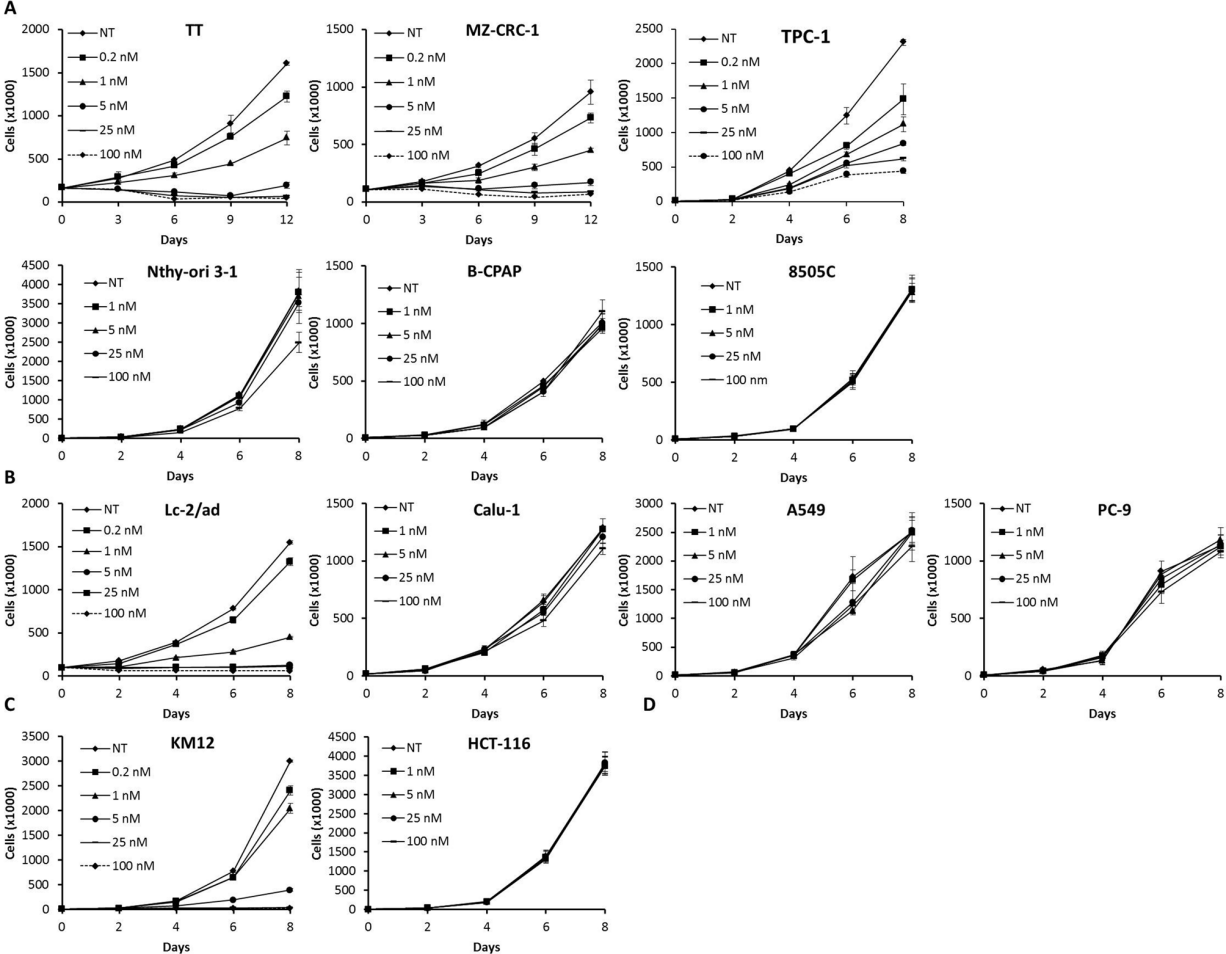
The indicated cell lines were incubated with vehicle (NT: not treated) or increasing concentrations of Pz-1 and counted at the indicated time points. Data are the mean ± SD of a single experiment performed in triplicate. Number of cells plated at Day 0: TT (200,000/well); MZ-CRC-1 and Lc-2/ad (100,000/well); Nthy-ori-3-1, 8505C, TPC-1 and HCT-116 (5,000/well); B-CPAP, A549, Calu-1, PC-9 and KM12 (10,000/well).

We analysed cell proliferation of RET- and TRKA-positive cancer cells using Pz-1 doses ranging from 0.2 to 100 nM. As shown in Fig. 3A,B, proliferation of RET-positive MTC (TT and MZ-CRC-1), PTC (TPC-1) and NSCLC (Lc-2/ad) cells started to be affected already at 0.2 nM Pz-1. In all these cells, the Pz-1 IC_50_ dose for proliferation was around 1 nM (Supplementary information, Table S1), a dose that was consistent with that required to hinder the RET kinase (Fig. 2). Similarly, proliferation of TPM3-TRKA-positive CRC (KM12) cells was inhibited by Pz-1 with an IC_50_ dose of around 1.6 nM (Fig. 3C and Supplementary information, Table S1) (Fig. 2). Instead, the proliferation of RET- and TRKA-negative cells was virtually left unaffected by up to 100 nM (B-CPAP, 8505C, A549, PC-9, Calu-1, HCT-116) or up to 25 nM (Nthy-ori-3-1) Pz-1 (Fig. 3 and Supplementary information, Table S1). Inhibition of RET (TT, MZ-CRC-1, TPC-1, Lc-2/ad) or TRKA (KM12) positive cells was also confirmed by MTT assay, in which cells were treated with a single dose of Pz-1 and analysed after 48 and 72 h. With this assay, 1 nM was able to modestly reduce RET or TRKA cell metabolic activity; the highest effect was reached at 10 nM (Supplementary information, Figure S5).

In order to expand the analysis of the effect of Pz-1 on a larger set of cancer cell lines, the NCI 60 five-dose screen was performed. As shown in Supplementary information Figure S6, KM12 was the only cell line displaying strong growth inhibition at a low (2 digit) nM dose, in accordance with the fact that this is the only cell line in the panel driven by a TRK-derived oncogene (no RET mutant cell line is included in the panel). No significant lethality was observed, thus suggesting that Pz-1 achieved principally a cytostatic rather than cytotoxic effect. At high Pz-1 doses, growth inhibition (> 100 nM) or lethality (> 1 µM) was also noted in some additional cell lines (Supplementary information, Figure S6).

**Figure 4:**
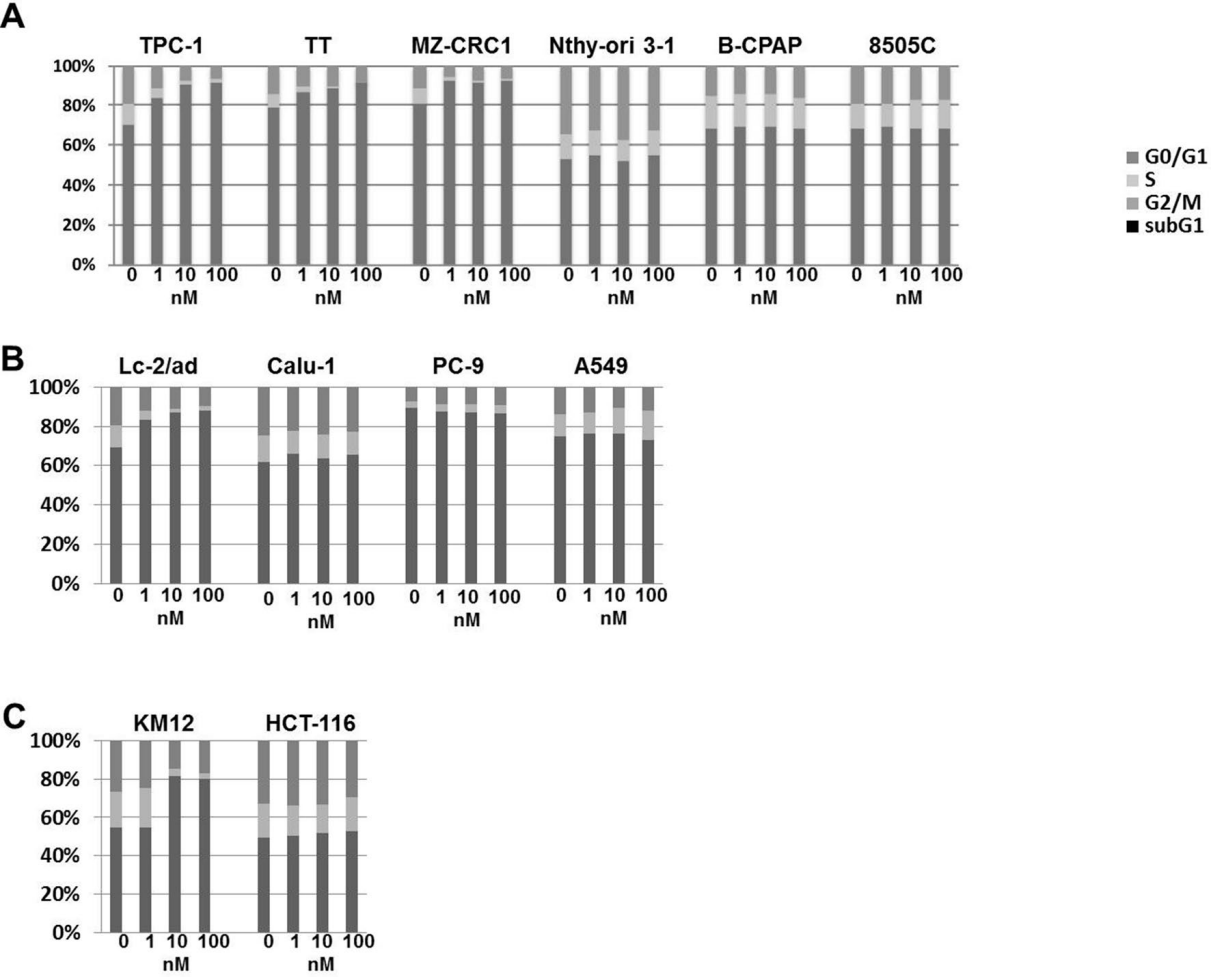
The indicated cell lines were incubated with increasing concentrations of Pz-1 for 48 h and then analysed by cytofluorimetry for DNA content. The bar graphs indicate the percentage of cells in G0/G1, S, G2/M and sub-G1 for each cell line.

We analysed cell cycle distribution upon treatment with 1, 10 and 100 nM Pz-1 for 48 h. As shown in Fig. 4, in RET-driven (TPC-1, TT, MZ-CRC-1, Lc-2/ad) cells, 1 nM dose of Pz-1 was able to cause a reduction of the percentage of cells in S and G2/M phases and an increase of cells in G0/G1 phase; the maximum effect on cell cycle arrest was achieved at 10 nM. In RET-negative (B-CPAP, 8505C, Nthy-ori-3-1, A549, PC-9 and Calu-1) cells, the cell cycle distribution was basically unaffected up to 100 nM concentration. Similarly, TRKA transformed KM12 cells, but not TRKA-negative HCT-116 cells, displayed a strong reduction of S and G2/M phases at 10 nM of Pz-1. No detectable subG1 fraction increase was noted, pointing to a primarily cytostatic effect of the drug.

### Pz-1 inhibits tumourigenicity of RET and TRKA driven cells

We previously reported that Pz-1 was able to abrogate tumourigenicity of RET C634Y-transformed NIH3T3 fibroblasts. Probably because of its anti-angiogenic activity, mediated by VEGFR2 inhibition, Pz-1 was also able to reduce growth of HRAS G12V-NIH3T3 tumours, although to a lower extent compared to RET-driven ones^55^.

**Figure 5:**
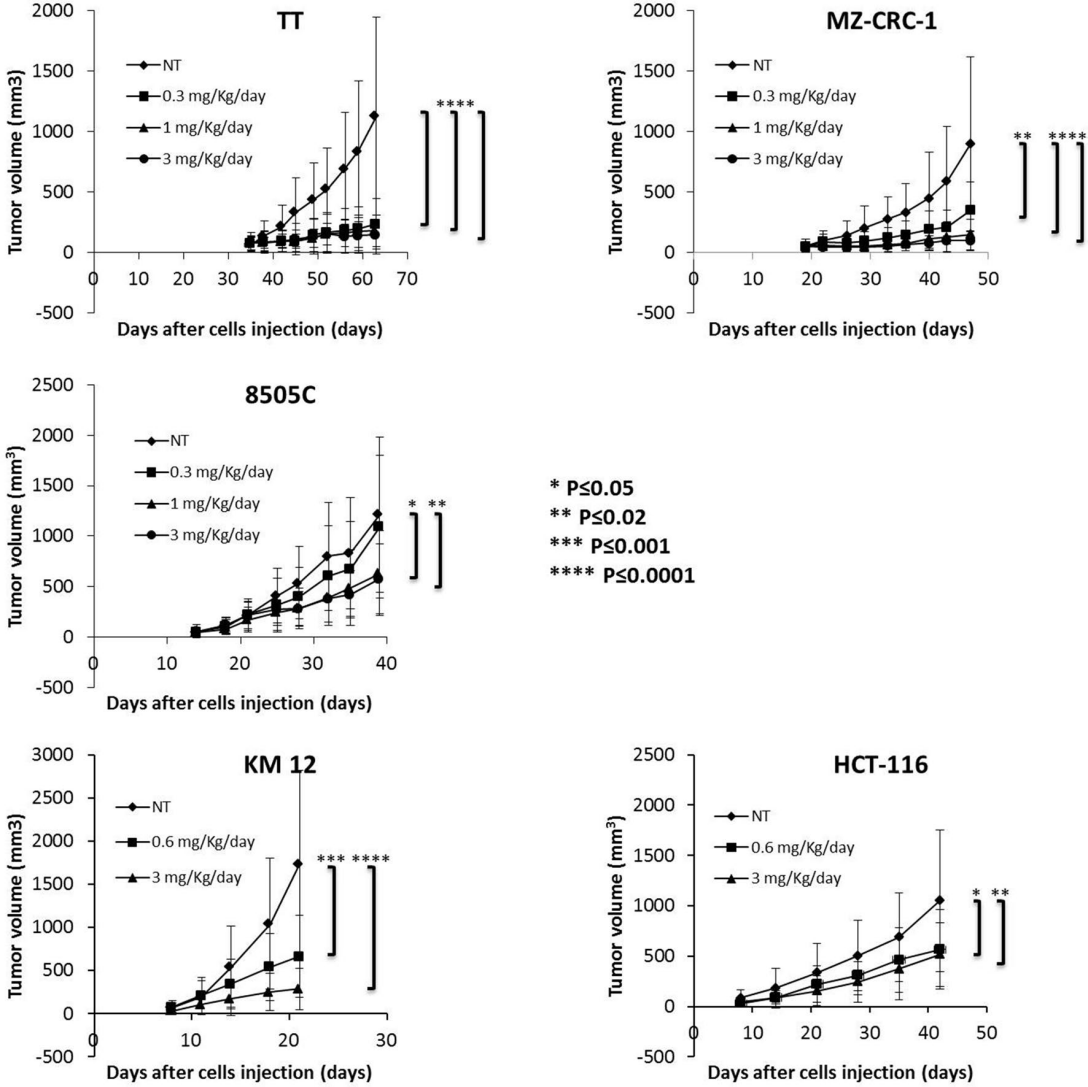
The indicated human cancer cell lines were inoculated subcutaneously into both flanks of either SCID (TT, MZ-CRC-1 and 8505C) or nu/nu (KM12 and HCT-116) mice. When tumours were visible, animals were randomly assigned to receive Pz-1 or vehicle for the indicated time points by oral gavage. Average size ± SD of tumours is reported.

Here, we tested activity of Pz-1 on xenografts of human cancer cell lines driven by the relevant kinases. To this aim, RET positive (TT and MZ-CRC-1) and negative (8505C) thyroid cancer cell lines and TRKA positive (KM12) and negative (HCT-116) CRC cell lines were injected in immunocompromised mice. Animals were subjected to oral administration of Pz-1 (0.3, 1.0, 3.0 in thyroid or 0.6 and 3 mg/kg in colorectal xenografts) or vehicle and tumour growth was measured (Fig. 5). In thyroid cancer models, 0.3 mg/Kg Pz-1 was able to reduce growth of TT and MZ-CRC-1 cells xenografts (< 20% and 36% of the vehicle control, respectively), and 1 mg/Kg Pz-1 virtually abrogated (15% of the vehicle) tumour growth. Instead, 0.3 mg/Kg Pz-1 did not exert any detectable inhibition of control 8505C tumours and only 1 mg/Kg and 3 mg/kg doses were able to significantly reduce (50% of the vehicle control) these tumours. In CRC models, 0.6 mg/Kg and 3 mg/Kg doses were able to reduce growth of KM12 cells xenografts to 35% and 15% of the control, respectively. The same doses had a smaller effect (60% reduction at 0.6 mg/Kg and 50% reduction at 3 mg/Kg) on TRK-negative HCT-116 xenografts.

Thus, though some general anti-tumour effect was noted, possibly mediated by inhibition of angiogenesis (VEGFR2), Pz-1 efficacy correlated with the positivity for RET and TRKA oncoproteins. Accordingly, in vivo target inhibition (IVTI) confirmed the ability of Pz-1 to reduce phosphorylation of RET and TRKA, and some downstream signalling targets, at the doses used for tumour growth inhibition (Supplementary information, Figure S7). Noteworthy, at the same doses, VEGFR2 inhibition was also detected pointing to a mixed driver kinase/angiogenic inhibitory effect of the drug (Supplementary information, Figure S7). 8505C and HCT116 tumours, treated with 0.3, 1 or 3 mg/Kg Pz-1, displayed inhibition of VEGFR2 but not of SHC, MAPK or PLCγ (Supplementary information, Figure S8). Therefore, VEGFR inhibition, though not sufficient to blunt intracellular signalling, may be involved in the reported inhibition of RET/TRKA-negative tumours.

To correlate RET inhibition to drug plasma concentration, we harvested MZ-CRC-1-induced tumour tissues and plasma after 2, 4, 8 and 24 h following the last dose of Pz-1. As shown in Supplementary information, Figure S9, Pz-1 levels in plasma were dose- and time-dependent, displaying the highest concentrations at 2 h and at 3 mg/Kg. Up to 8 h, at all the doses, drug concentration remained > 10 nM, a value higher than that needed to reach a complete RET inhibition in cell-based assays. Accordingly, RET inhibition was noted at this time point (Supplementary information, Figure S9). After 24 h, when the compound became undetectable, RET phosphorylation was fully restored.

### Pz-1 efficacy against RET and TRKA mutants mediating TKI resistance

**Figure 6:**
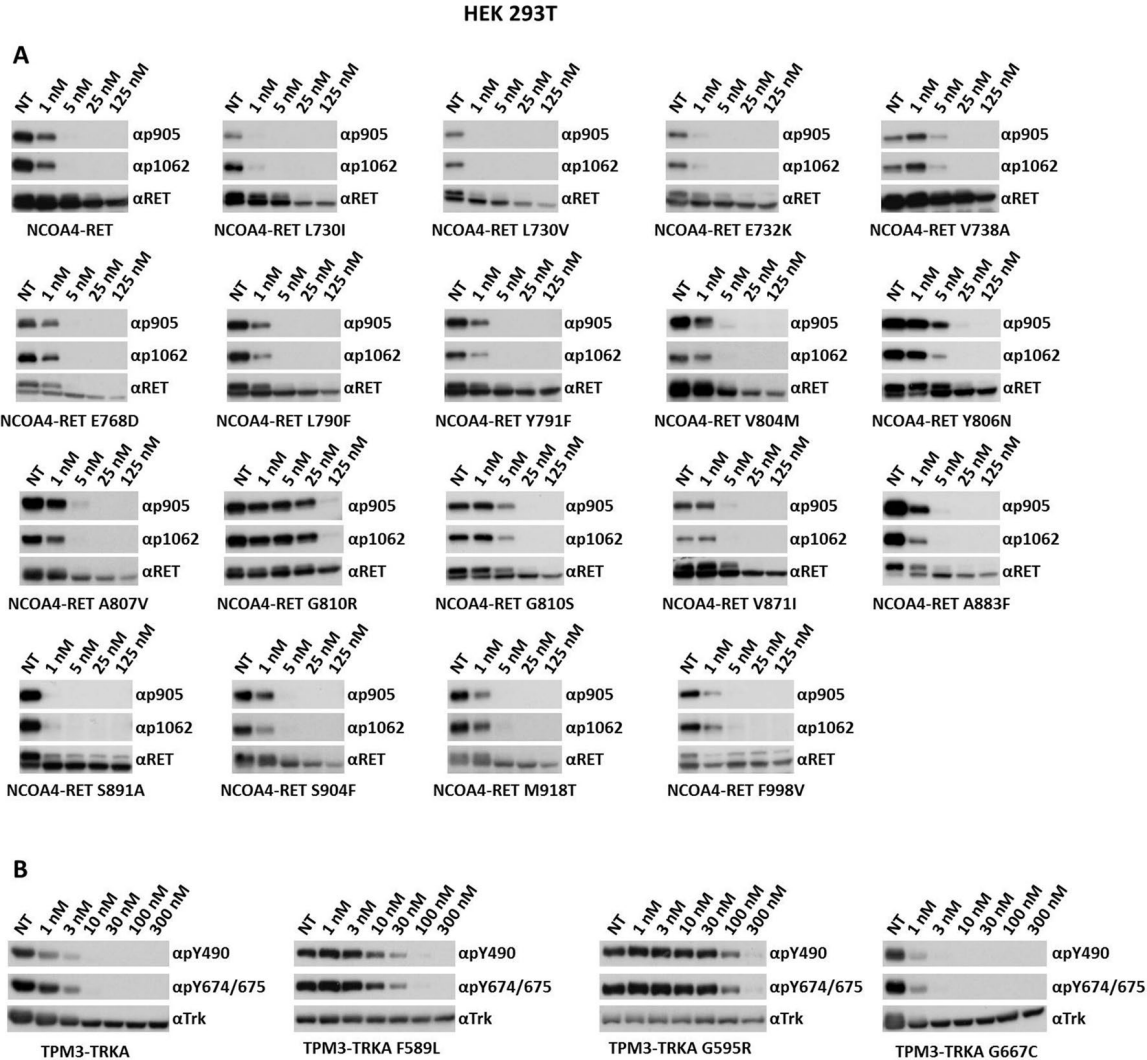
Serum-starved HEK 293 T cells transiently transfected with the indicated TPM3-TRKA and NCOA4-RET mutants were treated for 2 h with increasing concentrations of Pz-1. Total cell lysates (50 μg) were subjected to immunoblotting with the indicated anti-phospho TRK, and anti-phospho RET antibodies. The blots were normalized using anti-TRK and anti-RET antibodies.

We screened RET^3,46–54^ or TRKA^44,45^ single aminoacid mutants, either shown to display resistance to several TKIs or associated to cancer, for their sensitivity to Pz-1. The various mutations were cloned in the backbone of NCOA4-RET or TPM3-TRKA and transiently expressed in HEK 293 T cells; RET and TRKA tyrosine phosphorylation was measured upon 2 h treatment with different Pz-1 doses (Fig. 6). With the notable exception of G810 mutants, all the tested RET mutants remained sensitive to Pz-1 inhibition, including mutants in the kinase hinge (Y806N), roof of the solvent front (L730I/V), glycine-rich loop (E732K), β2 strand (V738A) and activation loop (S904F). As previously reported, mutations at the gatekeeper residue (V804M) did not impair Pz-1 activity^55^. In contrast, mutations of G810, in the solvent-exposed region, caused > 5 (G810S) or > 25 (G810R) fold resistance to Pz-1. In the case of TRKA, mutation in the DFG motif (G667C) did not affect sensitivity to Pz-1, while gatekeeper mutations (F589L) reduced by about 10 folds drug sensitivity. As in the case of RET, solvent front TRKA mutation (G595R) mediated strong resistance (> 30 fold) to Pz-1 (Fig. 6).

### Pz-1 anti-neoplastic activity is dependent on RET and TRKA inhibition

We stably expressed NCOA4-RET and TPM3-TRKA in NIH3T3 fibroblasts and measured Pz-1 effects on proliferation and phosphorylation. In this model system, Pz-1 was about 5 folds more active against NCOA4-RET than TPM3-TRKA (Supplementary information, Figure S10). Then, we used this system to verify G810R (for RET) and G595R (for TRKA) resistance to Pz-1. Pz-1 was virtually not effective against the two mutant kinases up to 30 nM concentration; accordingly, NCOA4-RET G810R and TPM3-TRKA G595R cells displayed a strong increase of Pz-1 IC_50_ for cell proliferation inhibition (Supplementary information, Figure S10).

**Figure 7:**
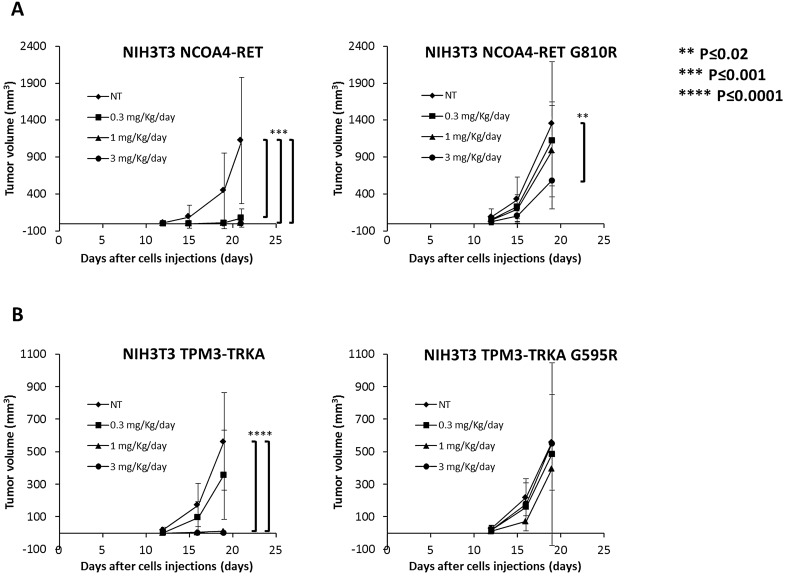
The indicated RET- and TRKA-transformed NIH3T3 cells were inoculated subcutaneously into both flanks of nu/nu mice. After four days, mice were randomly assigned to receive Pz-1 (0.3, 1 or 3 mg/Kg) by oral gavage or left untreated. Average size ± SD of tumours is reported.

Thus, we used NCOA4-RET G810R and TPM3-TRKA G595R NIH3T3 xenografts to address RET and TRKA dependency of Pz-1 anti-neoplastic activity in vivo. Treatment with 0.3 mg/Kg of Pz-1 was able to completely block NIH3T3 NCOA4-RET but not TPM3-TRKA tumour growth. Complete tumour growth inhibition was seen with 1 and 3 mg/Kg Pz-1 in both models. Importantly, NCOA4-RET G810R tumour growth remained virtually unaffected by 0.3 and 1 mg/Kg doses; only 3 mg/Kg treatment was able to reduce tumour volume by about 50%. TPM3-TRKA G595R tumour growth remained unaffected by Pz-1 at all the doses (Fig. 7). At the end of tumour growth experiment, phosphorylation of NCOA4-RET G810R and TPM3-TRKA G595R confirmed their strong resistance to Pz-1, with only NCOA4-RET G810R showing some rate of inhibition at the highest used dose (3 mg/Kg), possibly explaining the partial tumour growth inhibition (Supplementary information, Figure S11). VEGFR2 inhibition was already detectable at 0.3 mg/kg in both Pz-1 sensitive and resistant RET or TRKA-expressing tumours; therefore, although angiogenesis inhibition may contribute to the anti-tumour effect of the drug, alone it was not sufficient to blunt the growth of these highly aggressive tumours in the presence of Pz-1 resistant RET or TRKA mutants (Supplementary information, Figure S11).

We used the same model system to further address dose-dependency and duration of RET and TRKA inhibition by Pz-1. In NIH3T3 NCOA4-RET tumours, 0.3, 1 and 3 mg/kg doses were all able to inhibit RET up to 12 h, with RET phosphorylation being partially restored after 24 h (Supplementary information, Figure S12). Pz-1 inhibitory effect had a shorter duration in TPM3-TRKA tumours; thus, TRKA phosphorylation was fully restored after 8 h of treatment with 0.3 mg/Kg Pz-1, and after 12 h of treatment with 1 and 3 mg/Kg when it returned to levels even higher than baseline (Supplementary information, Figure S12). Such a faster kinetics correlated with the reduced Pz-1 efficacy in TPM3-TRKA compared to NCOA4-RET xenografts (Fig. 7).

## Discussion

Here, we report that Pz-1, a RET and VEGFR2 inhibitor^55^, potently inhibit also TRKs kinases. RET and TRK mutations are in general mutually exclusive in cancer, with the exception of rare instances in which a secondary TRK mutation may bypass RET inhibition^59^. Thus, the Pz-1 inhibitory profile suggested that it may prove useful in cancer driven either by RET or TRKA. Here, we tested Pz-1 anti-neoplastic activity in relevant (thyroid, NSCLC, and CRC) human RET or TRKA-driven cancer models. In cell-based assays, Pz-1 selectively inhibited, in the low nM range, proliferation of cancer cell lines only when positive for RET or TRK-derived oncogenes, with negligible effects in cells carrying different driver oncogenes (RAS, BRAF, EGFR). When tested against a panel of RET or TRKA mutants displaying resistance to other TKI, Pz-1 was remarkably active against most of the tested mutations, including RET Y806N, L730I/V, E732K, and V738A that featured resistance to the new generation inhibitors pralsetinib, selpercatinib or both^46–54^. Moreover, Pz-1 maintained activity against TRKA G667C that mediated resistance to entrectinib and larotrectinib^44,45^. However, mutations of G810 and G595, in the RET or TRKA solvent-exposed region, respectively, caused > 25 (NCOA4-RET G810R) and > 30-fold (TPM3-TRKA G595R) resistance resistance to Pz-1. Noteworthy, G810S increased half-maximal inhibitory Pz-1 dose for NCOA4-RET by less than 1 log (around 5 nM) (Fig. 6 and data not shown), therefore with an IC_50_ still compatible with its inhibition in vivo. In this frame, TPX-0046, a RET/SRC inhibitor that is currently in Phase 1 testing, has demonstrated preclinical potency against RET G810 mutations^60^.

Cell-based Pz-1 effects were translated into potent in vivo anti-tumour activity against RET or TRKA driven xenograft models. However, at a variance from the in vitro setting, in vivo Pz-1 attenuated growth of RET/TRKA-negative cancer models, as well. This RET/TRKA independent activity was possibly mediated by its (anti-VEGFR2) anti-angiogenic activity due to VEGFR2 inhibition, though at this stage is not possible to exclude other drug targets. Thus, to address the importance of RET and TRKA inhibition for the anti-tumour effects, we took advantage of solvent front RET (G810R) or TRKA (G595R) mutants that display resistance to Pz-1. Compound efficacy against NIH3T3 cells transformed by NCOA4-RET G810R and TPM3-TRKA G595R was strongly attenuated with respect to NCOA4-RET and TPM3-TRKA, indicating that RET and TRKA inhibition is necessary, albeit not necessarily sufficient, for Pz-1 to achieve its anti-neoplastic effect. In addition, at the remarkably low dose at which it displays anti-neoplastic activity (0.3 mg/kg), Pz-1 was able to inhibit both RET and TRKA.

In conclusion, these findings point to Pz-1 as a promising anti-cancer agent towards either RET or TRKA driven cancers, though, in a clinical setting its VEGFR2 activity that probably collaborates with RET and TRKA activity, has to be kept under check because it may be responsible of dose-limiting toxicity.

## Supplementary Information

Supplementary Information

Supplementary Information.

## Abbreviations

RTK: Receptor tyrosine kinase
TKI: Tyrosine kinase inhibitor
PTC: Papillary thyroid carcinoma
MTC: Medullary thyroid carcinoma
NSCLC: Non-small-cell lung cancer
CRC: Colorectal cancer
PFS: Progression free survival
TRK: Tropomyosin receptor kinase
VEGFR2: Vascular endothelial growth factor receptor 2
RET: Rearranged during transfection
MASC: Mammary analogue secretory carcinoma
