# Supplementary material for: Targeted activity of the small molecule kinase inhibitor Pz-1 towards RET and TRK kinases
Source: Sci Rep. 2021 Aug 9;11:16103. doi: 10.1038/s41598-021-95612-4 (PMC8352932; doi:10.1038/s41598-021-95612-4)
Supplement: Supplementary file 1 — Supplementary Information [file 41598_2021_95612_MOESM1_ESM.docx]

**Supplementary information**

**Targeted activity of the small molecule kinase inhibitor Pz-1 towards RET and TRK kinases**

**Marialuisa Moccia et al.**

**
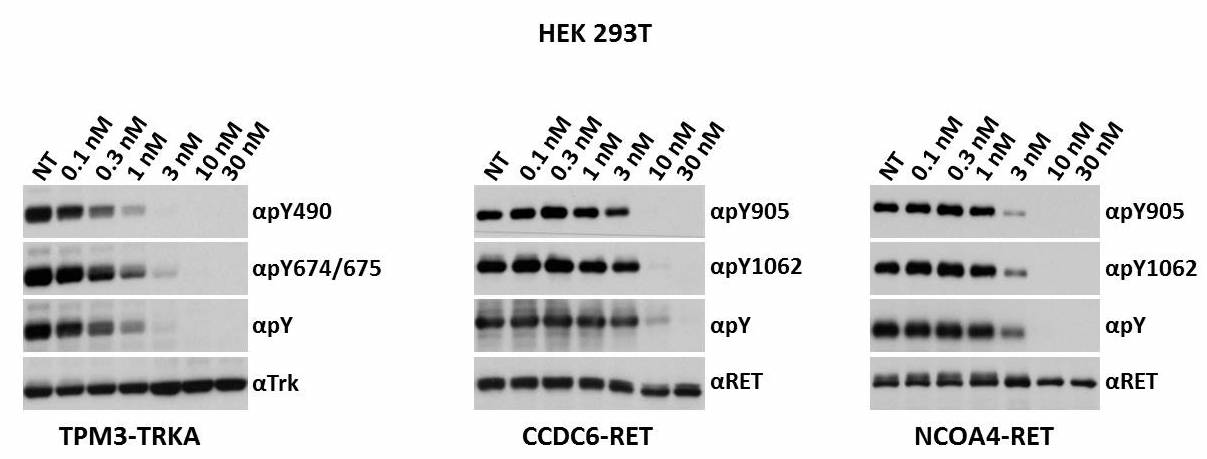
**

**Figure S1.** Serum-starved HEK 293T cells transiently transfected with the indicated TRKA and RET rearranged mutants were treated for 2 hrs with increasing concentrations of Pz-1. Total cell lysates (50 μg) were subjected to immunoblotting with the indicated anti-phospho TRK, anti-phospho RET and anti-phosphotyrosine (αpTyr) antibodies. The blots were normalized using anti-TRK and anti-RET antibodies.


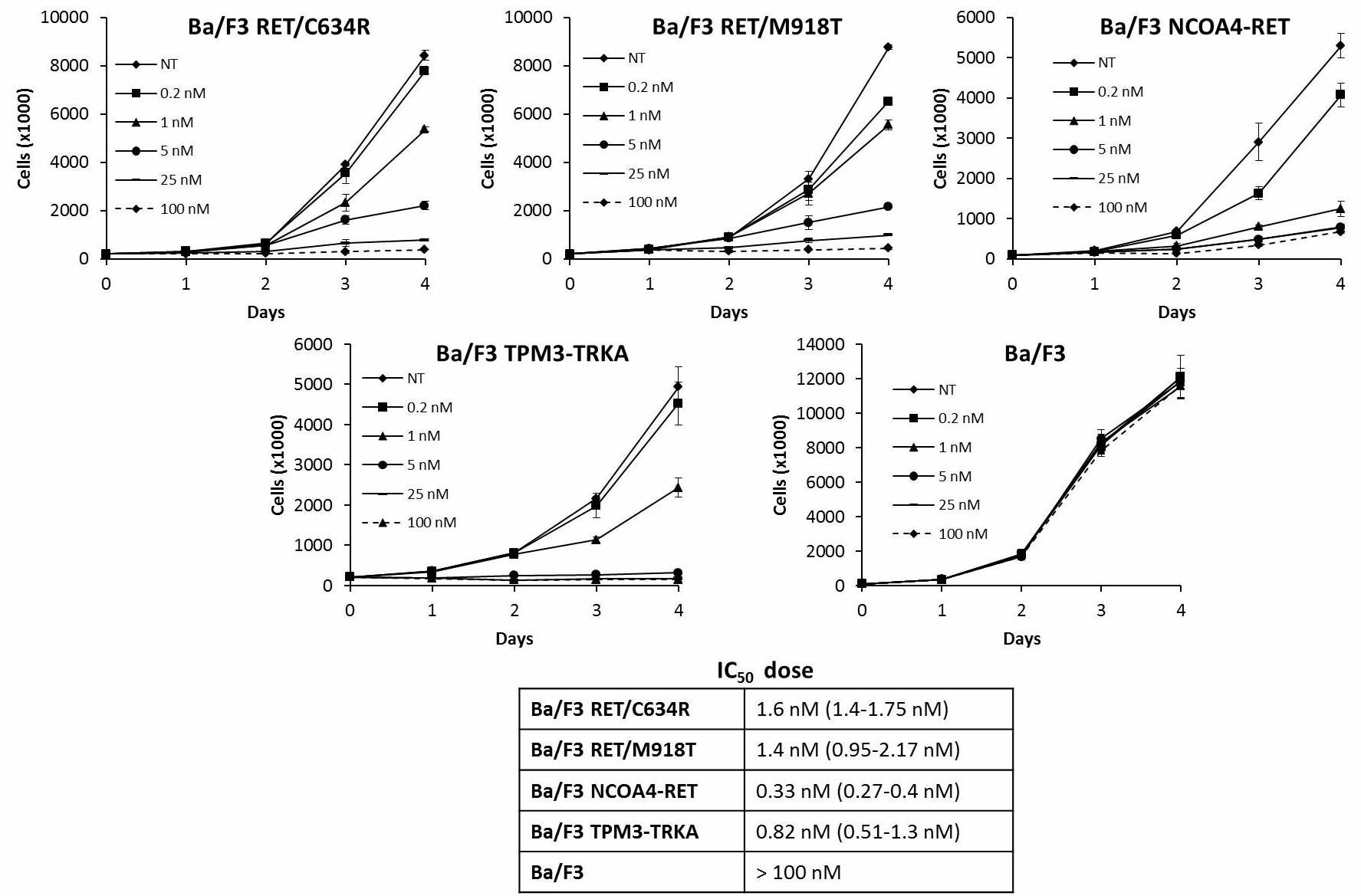


**Figure S2.** Ba/F3 cells, stably transfected with the indicated RET- or TRKA-derived oncogenes, were incubated with vehicle (NT: not treated) or the indicated concentrations of Pz-1 and counted daily for 4 consecutive days. Data are the mean ± SD of a single experiment performed in triplicate. Parental Ba/F3 cells were used as control.


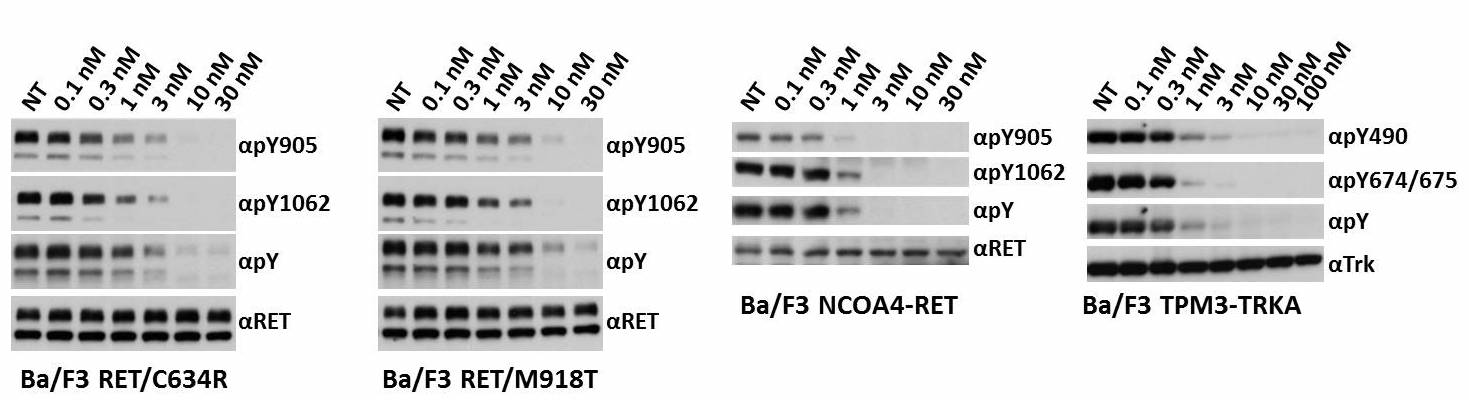


**Figure S3.** Ba/F3 cells, stably transfected with the indicated RET- or TRKA-derived oncogenes, were serum starved and treated for 2 hours with increasing concentrations of Pz-1. Total cell lysates (50 μg) were subjected to immunoblotting with the indicated antibodies.


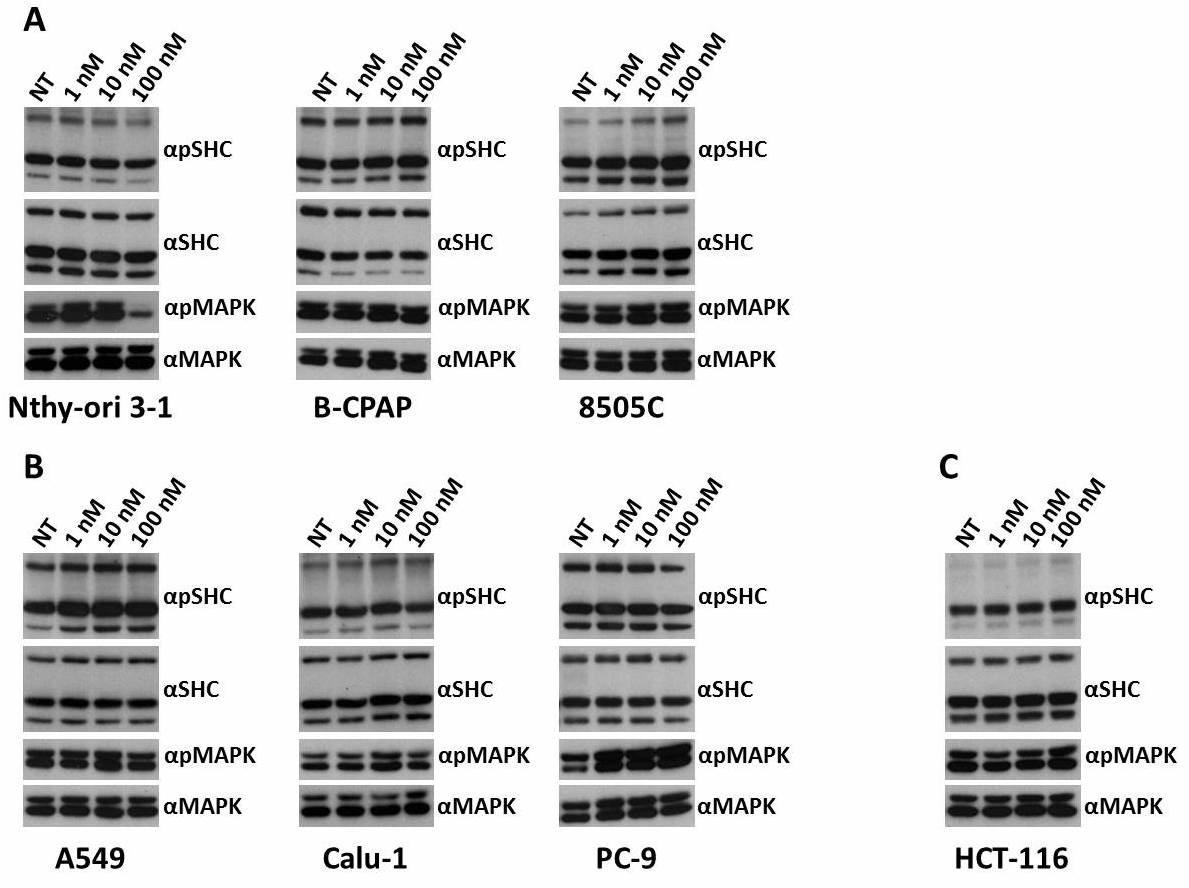


**Figure S4.** The indicated cell lines were serum-starved and treated for 2 hours with increasing concentrations of Pz-1. Total cell lysates (50 μg) were subjected to immunoblotting with the indicated antibodies.

**
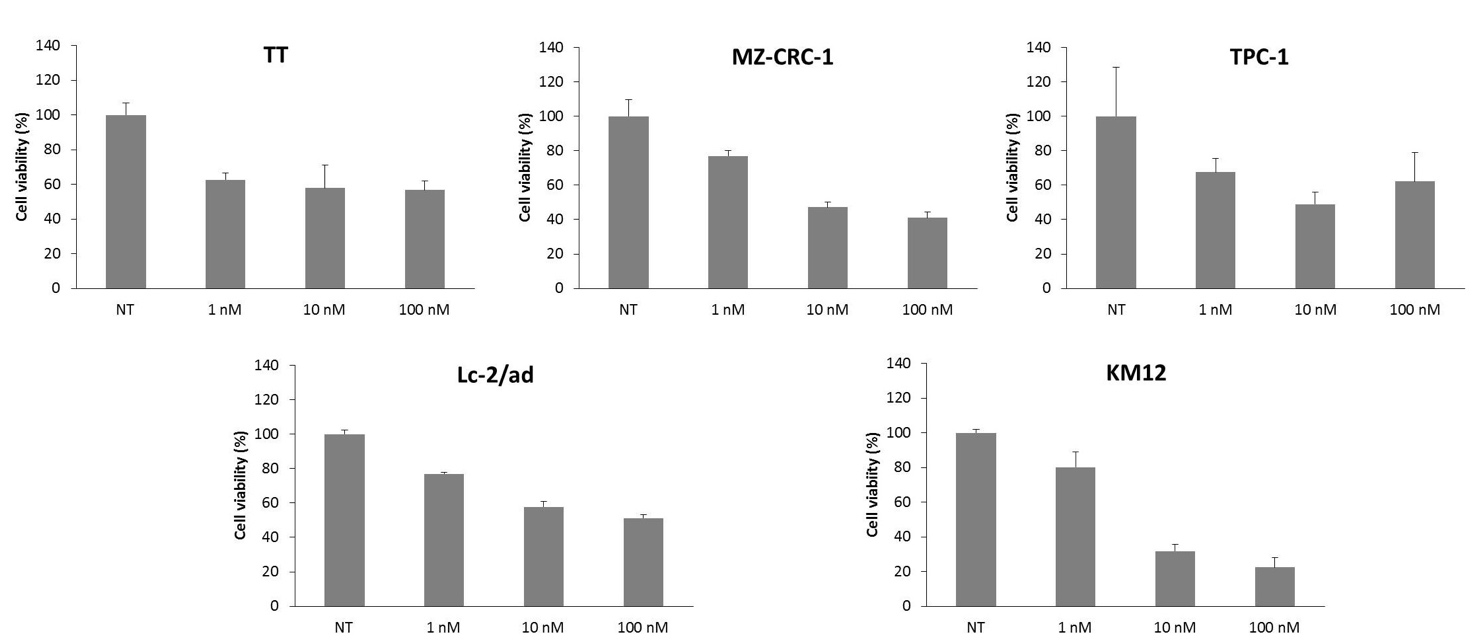
**

**Figure S5.** The indicated cell lines were treated for 72 (TT and MZCRC-1) or 48 (TPC-1, Lc-2/ad and KM12) hours with increasing concentrations of Pz-1 and then subjected to MTS assay. Data are the mean ± SD of a single experiment performed in triplicate.

**
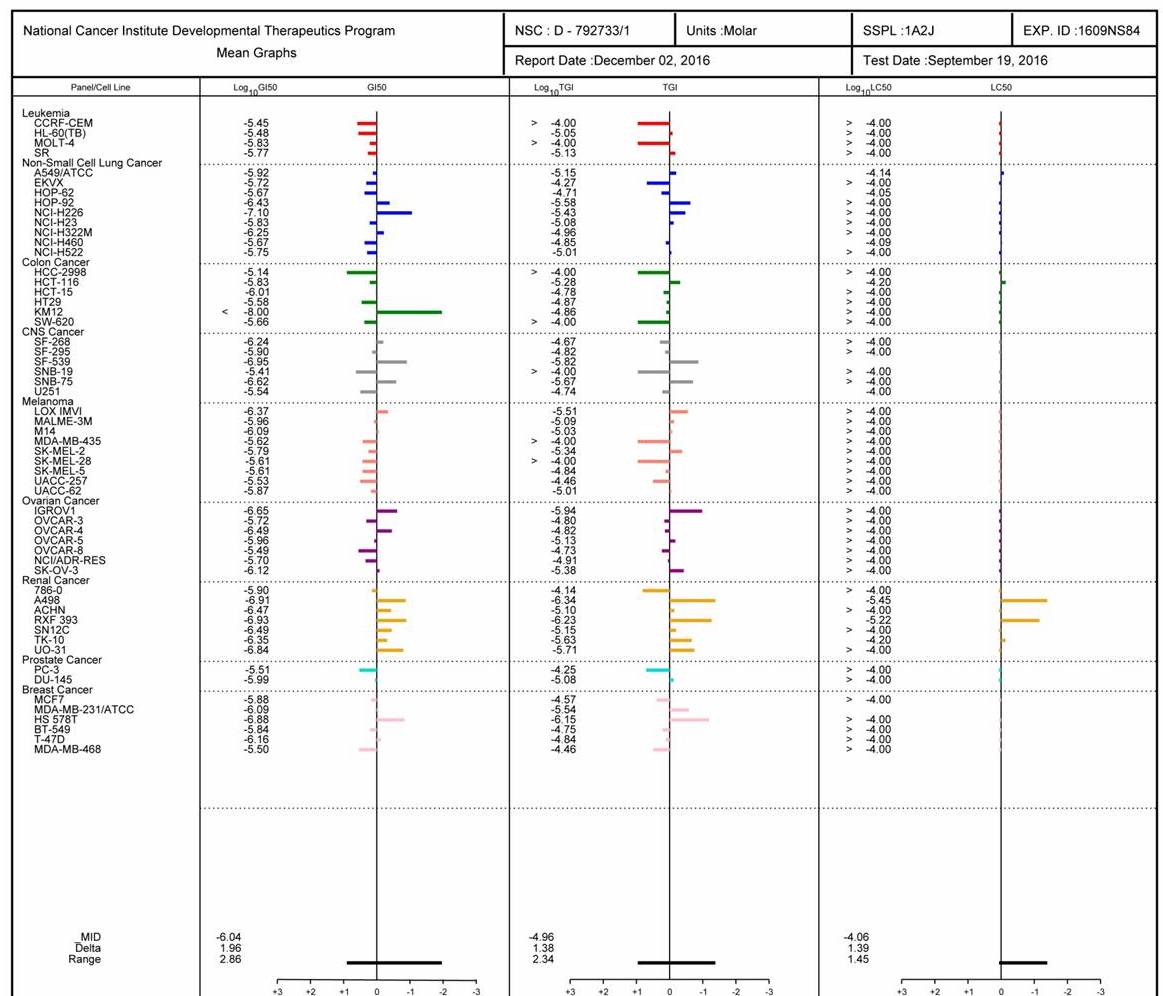
**

**Figure S6.** NCI 60 five-dose screen using Pz-1 at doses ranging from 1 to 1 x 10^-8^ M. GI50: 50% growth inhibition. TGI: total growth inhibition. LC50: 50% lethal concentration.


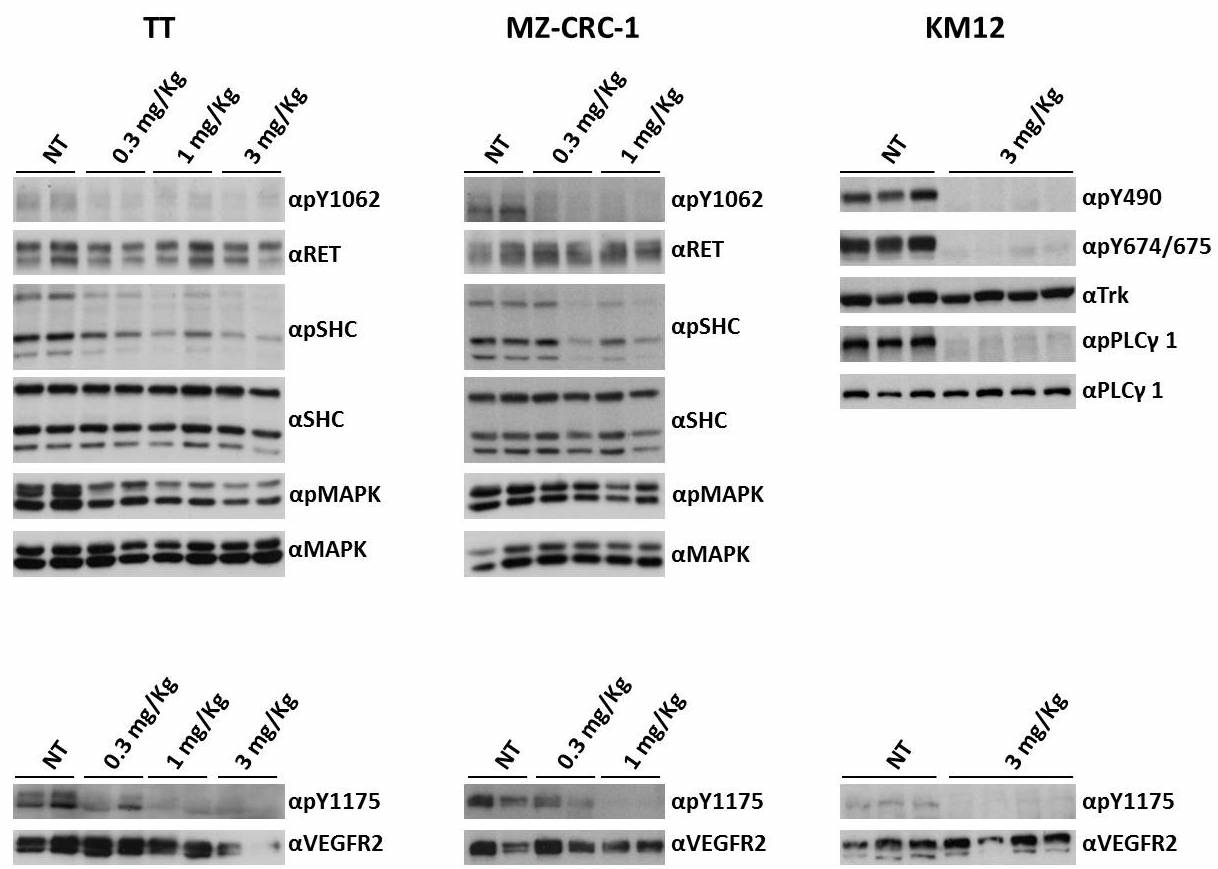


**Figure S7.** TT (1.5 x 10^7^), MZ-CRC-1 (1 x 10^7^) and KM12 (1 x 10^7^) cells were inoculated subcutaneously into the right and left dorsal portions of either SCID (TT and MZ-CRC-1) or nu/nu mice (KM12). When tumours reached approximately ~400-500 mm^3^, mice were treated by oral gavage with 2 doses (0, 24; 2 mice/dose) of Pz-1, or left untreated (NT); protein lysates were extracted from tumours harvested 3 hrs after the last dose. Upper panels: Total lysates (50 μg) from 2-4 representative tumours for each xenografted cell line and for each dose were immunoblotted with the indicated antibodies. Lower panels: Total lysates (1 mg) from 2-4 representative tumours for each xenografted cell line and for each dose were subjected to VEGFR2 immunoprecipitation followed by Western blotting with anti-phospho-VEGFR2 antibody (αpY1175). The blots were normalized using anti-VEGFR2 (αVEGFR2).


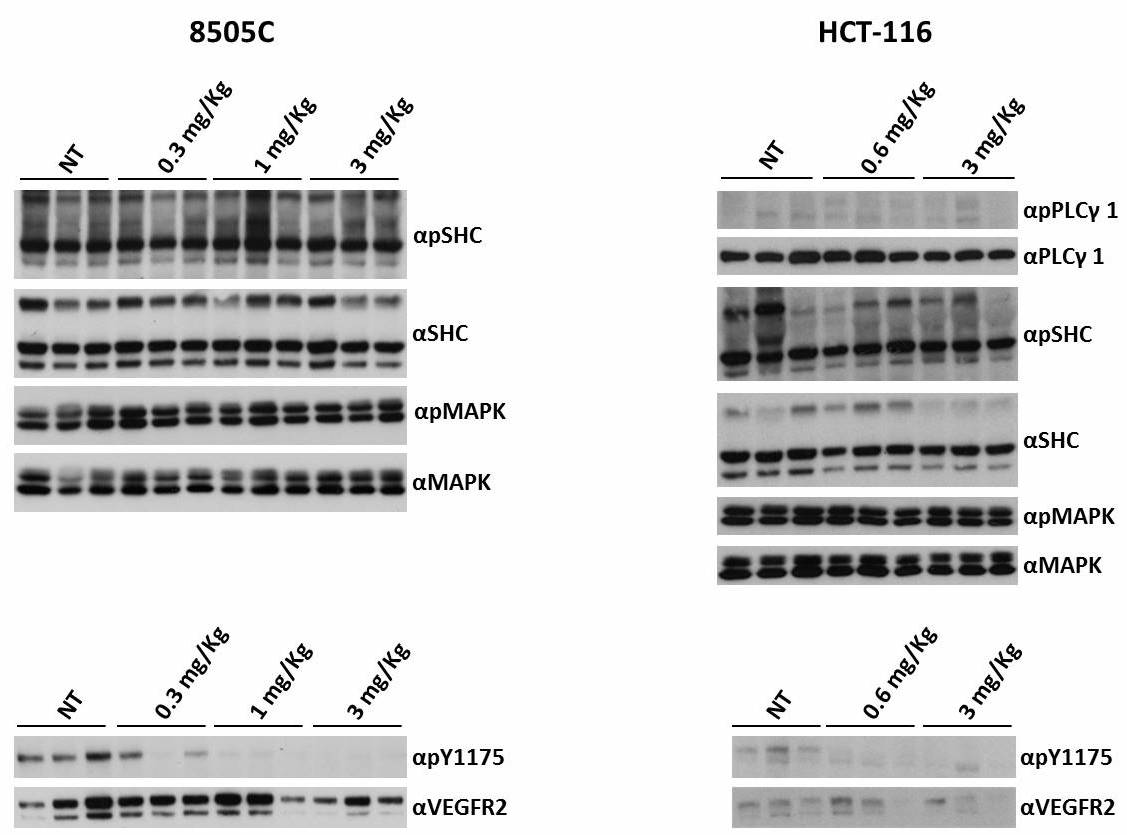


**Figure S8.** 8505-C (1 x 10^7^) and HCT116 (1 x 10^7^) cells were inoculated subcutaneously into the right and left dorsal portions of either SCID (8505-C) or nu/nu (HCT116) mice. When tumours reached approximately ~400-500 mm^3^, mice were treated by oral gavage with 2 doses (0, 24 hrs; 2 mice/dose) of Pz-1 at 0.3, 1 and 3 mg/Kg concentration, or left untreated (NT); protein lysates were extracted from tumours harvested 3 hrs after the last dose. Upper panels: Total lysates (50 μg) from 3 representative tumours for each xenografted cell line and for each dose were immunoblotted with indicated antibodies. Lower panels: Total lysates (1 mg) from 3 representative tumours for each xenografted cell line and for each dose were subjected to VEGFR2 immunoprecipitation followed by Western blotting with anti-phospho-VEGFR2 antibody (αpY1175). The blots were normalized using anti-VEGFR2 (αVEGFR2).


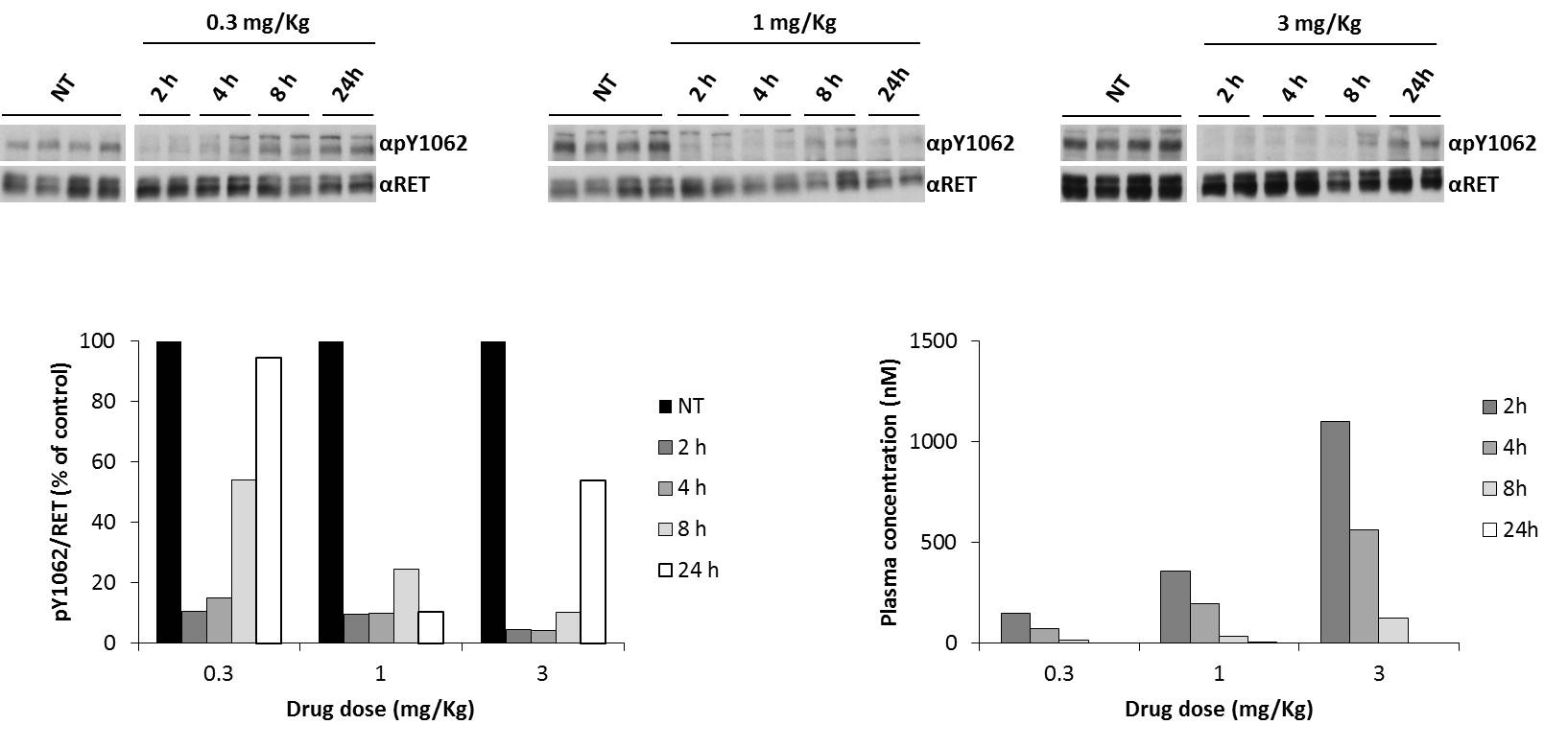


**Figure S9.** MZ-CRC-1 (1 x 10^7^) cells were inoculated subcutaneously into the right and left dorsal portions of SCID mice. When tumours reached approximately ~50 mm^3^, mice were treated by oral gavage with 1 dose (3 concentrations; 0.3, 1 and 3 mg/Kg; Pz-1 or vehicle (NT) for 28 consecutive days; plasma and tumours were harvested at indicated time points following the last dose. Upper panels: Protein lysates were extracted from tumours and total lysates (50 μg) from 2-4 representative tumours for each xenografted cell line and for each dose were immunoblotted with indicated antibodies. Lower panels: densitometric analysis of RET pY1062 phosphorylation vs total RET was performed using ImageJ software and mean values for each group were plotted (left); free plasma concentration was measured by LC-MS/MS and mean values for each group were plotted (right).

**
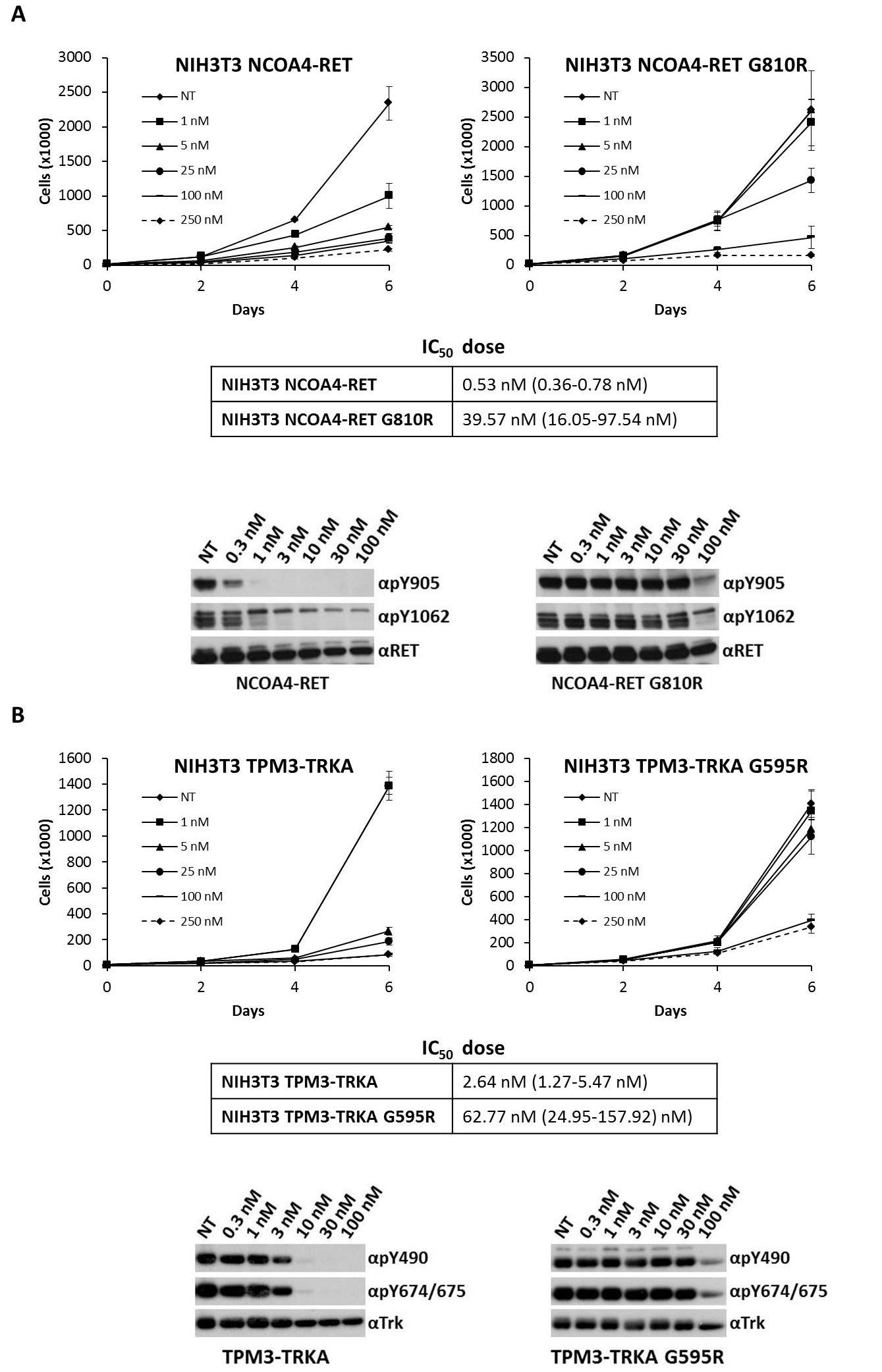
**

**Figure S10.** NIH3T3 cells stably transfected with NCOA4-RET, NCOA4-RET G810R, TPM3-TRKA and TPM3-TRKA G595R expressing vectors were incubated with vehicle (NT: not treated) or increasing concentrations of Pz-1 and counted at the indicated time points. Data are the mean ± SD of a single experiment performed in triplicate. In the table the IC_50_ doses of Pz-1 for each cell line are reported (A and B upper and central panels). Serum-starved NIH3T3 cells stably transfected with NCOA4-RET, NCOA4-RET G810R, TPM3-TRKA and TPM3-TRKA G595R expressing vectors were treated for 2 hrs with increasing concentrations of Pz-1. Total cell lysates (50 μg) were subjected to immunoblotting with the indicated anti-phospho TRK, and anti-phospho RET antibodies. The blots were normalized using anti-TRK and anti-RET antibodies (And B lower panels).


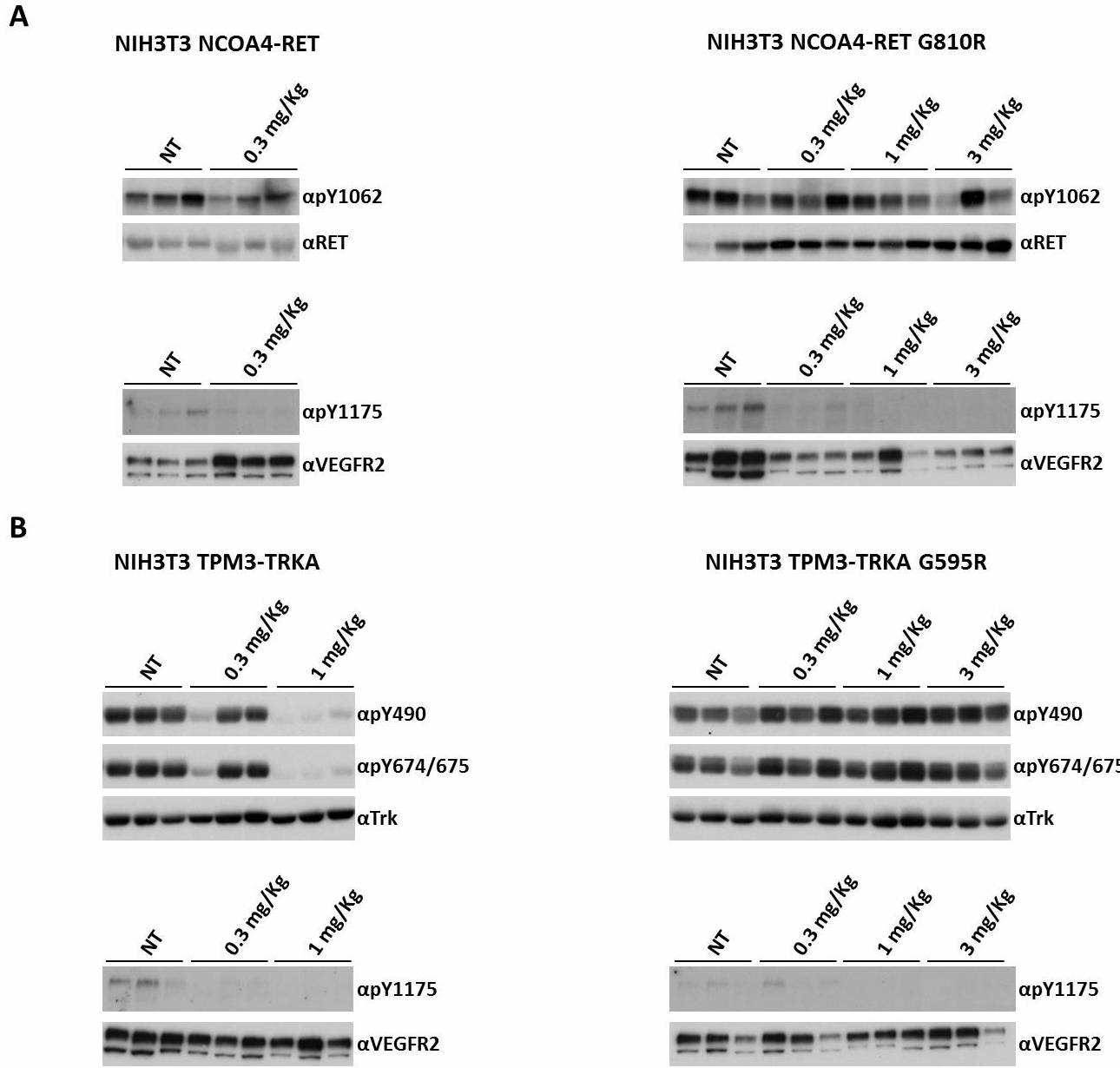


**Figure S11.** NIH3T3 NCOA4-RET, NCOA4-RET G810R, TPM3-TRKA and TPM3-TRKA G595R cells were inoculated subcutaneously into the right and left dorsal portions of nu/nu mice. Animals were randomly assigned to receive Pz-1 or vehicle control. After 4 days from cell injection, animals were treated with vehicle, 0.3, 1 or 3 mg/Kg/day Pz-1 by oral gavage for 16 (NCOA4-RET and NCOA4-RET G810R) or 17 consecutive days (TPM3-TRKA and TPM3-TRKA G595R). Total lysates (50 μg) from 3 representative tumours for each xenografted cell line and for each dose (with the exception of 1 and 3 mg/kg for NCOA4-RET and 3 mg/Kg for TPM3-TRKA since tumours did not grow) were immunoblotted with the indicated antibodies.


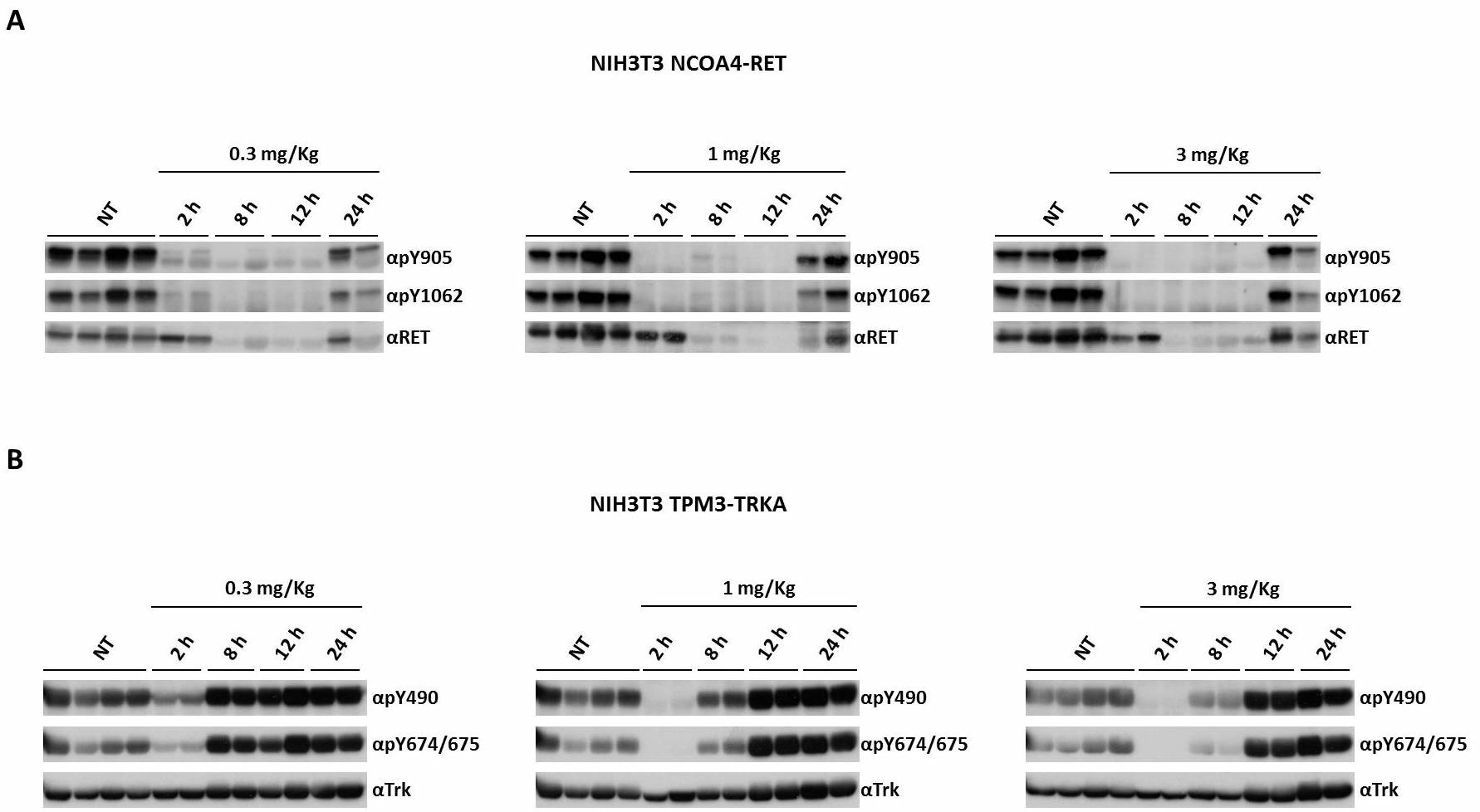


**Figure S12.** NIH3T3 NCOA4-RET and TPM3-TRKA cells were inoculated subcutaneously into the right and left dorsal portions of nu/nu mice. When tumours reached approximately ~400-500 mm^3^, mice were treated by oral gavage with 1 dose (3 concentrations; 0.3, 1 and 3 mg/Kg; 2 mice/concentration) of Pz-1, or left untreated (NT); tumours were harvested at indicated time points. Protein lysates were extracted from tumours and total lysates (50 μg) from 2-4 representative tumours for each xenografted cell line and for each dose were immunoblotted with the indicated antibodies.

**Table S1: Cell proliferation inhibitory effect exerted by Pz-1**

| Cell line | Origin* | Mutation | IC_50_ dose nM (95% CI) |
| --- | --- | --- | --- |
| TT | MTC | RET C634W | 0.77 (0.64-0.92) |
| MZ-CRC-1 | MTC | RET M918T | 0.72 (0.54-0.96) |
| TPC-1 | PTC | CCDC6-RET | 0.47 (0.33-0.67) |
| Nthy-ori-3-1 | SV40 | SV40 LT | > 100 |
| B-CPAP | PTC | BRAF V600E | > 100 |
| 8505C | ATC | BRAF V600E | > 100 |
| LC-2/ad | NSCLC | CCDC6-RET | 0.52 (0.37-0.74) |
| Calu-1 | NSCLC | KRAS G12C | > 100 |
| A549 | NSCLC | KRAS G12S | > 100 |
| PC-9 | NSCLC | HER1 delLREA | > 100 |
| KM12 | CRC | TPM3-TRKA | 1.66 (1.14-2.42) |
| HCT-116 | CRC | KRAS G38A | > 100 |

*MTC (medullary thyroid carcinoma); PTC (papillary thyroid carcinoma); ATC (anaplastic thyroid carcinoma); NSCLC (non-small cell lung carcinoma); CRC (colorectal carcinoma)

**Table S2: Primers used for site directed mutagenesis**

| **GENE** | **Mutation** | **PrimerS** |
| --- | --- | --- |
| **ret9** | L730I | 5'-ccaaattcgccttctcctatagtttttccaagaaccaag-3'  5'-cttggttcttggaaaaactataggagaaggcgaatttgg-3' |
|  | L730V | 5'-ccaaattcgccttctcctacagtttttccaagaaccaag-3'  5'-cttggttcttggaaaaactgtaggagaaggcgaatttgg-3' |
|  | E732K | 5'-ccacttttccaaattcgccttttcctagagtttttccaagaac-3'  5'-gttcttggaaaaactctaggaaaaggcgaatttggaaaagtgg-3' |
|  | V738A | 5'-gccgttgccttgaccgcttttccaaattcgcc-3'  5'-ggcgaatttggaaaagcggtcaaggcaacggc-3' |
|  | E768D | 5'-cctccccgagtgaccttcgagacctgc-3'  5'-GCAGGTCTCGAAGGTCACTCGGGGAGG-3' |
|  | L790F | 5'-ccacccacatgtcatcaaattctatggggcctgc-3'  5'-GCAGGCCCCATAGAATTTGATGACATGTGGGTGG-3' |
|  | Y791F | 5'-ccacccacatgtcatcaaattgtttggggcctgcag-3'  5'-CTGCAGGCCCCAAACAATTTGATGACATGTGGGTGG-3' |
|  | V804M | 5'-CGCTCCTCCTCATCATGGAGTACGCCAAATAC-3'  5'-GTATTTGGCGTACTCCATGATGAGGAGGAGCG-3' |
|  | Y806N | 5'-agccgtatttggcgttctccacgatgaggag-3'  5'-ctcctcatcgtggagaacgccaaatacggct-3' |
|  | A807V | 5'-cagggagccgtatttgacgtactccacgatgag-3'  5'-ctcatcgtggagtacgtcaaatacggctccctg-3' |
|  | G810R | 5'-gagtacgccaaataccgctccctgcgg-3'  5'-ccgcagggagcggtatttggcgtactc-3' |
|  | G810S | 5'-cccgcagggagctgtatttggcgtactcca-3'  5'-tggagtacgccaaatacagctccctgcggg-3' |
|  | V871I | 5'-ccaagtcccgatgaatgagcttcatctcggc-3'  5'-gccgagatgaagctcattcatcgggacttgg-3' |
|  | A883F | 5'-ggcagccagaaacatcctggtatttgaggggcggaa-3'  5'-TTCCGCCCCTCAAATACCAGGATGTTTCTGGCTGCC-3' |
|  | S891A | 5'-gaggggcggaagatgaagattgcggatttcggc-3'  5'-gccgaaatccgcaatcttcatcttccgcccctc-3' |
|  | S904F | 5'-ctcctcttcacgtagaaatcctcttcataaacatctcgggac-3'  5'-gtcccgagatgtttatgaagaggatttctacgtgaagaggag-3' |
|  | M918T | 5'-ggattcaattgccgtccatttaactggaatccgacc-3'  5'-ggtcggattccagttaaatggacggcaattgaatcc-3' |
|  | F998V | 5'-tgctgatgtccgcaaccaccggccttttgtc-3'  5'-gacaaaaggccggtggttgcggacatcagca-3' |
| **ntrk1** | F589L | 5'-ccccctgctcatggtcttagagtatatgcgg-3'  5'-ccgcatatactctaagaccatgagcaggggg-3' |
|  | G595R | 5'-agtatatgcggcacagggacctcaaccgc-3'  5'-gcggttgaggtccctgtgccgcatatact-3' |
|  | G667C | 5'-ggactggtggtcaagatttgtgattttggcatgagc-3'  5'-gctcatgccaaaatcacaaatcttgaccaccagtcc-3' |
