# Supplementary material for: Targeted activity of the small molecule kinase inhibitor Pz-1 towards RET and TRK kinases
Source: Sci Rep. 2021 Aug 9;11:16103. doi: 10.1038/s41598-021-95612-4 (PMC8352932; doi:10.1038/s41598-021-95612-4)
Supplement: Supplementary file 2 — Supplementary Information. [file 41598_2021_95612_MOESM2_ESM.pdf]

Figure 2 A
MZ-CRC-1

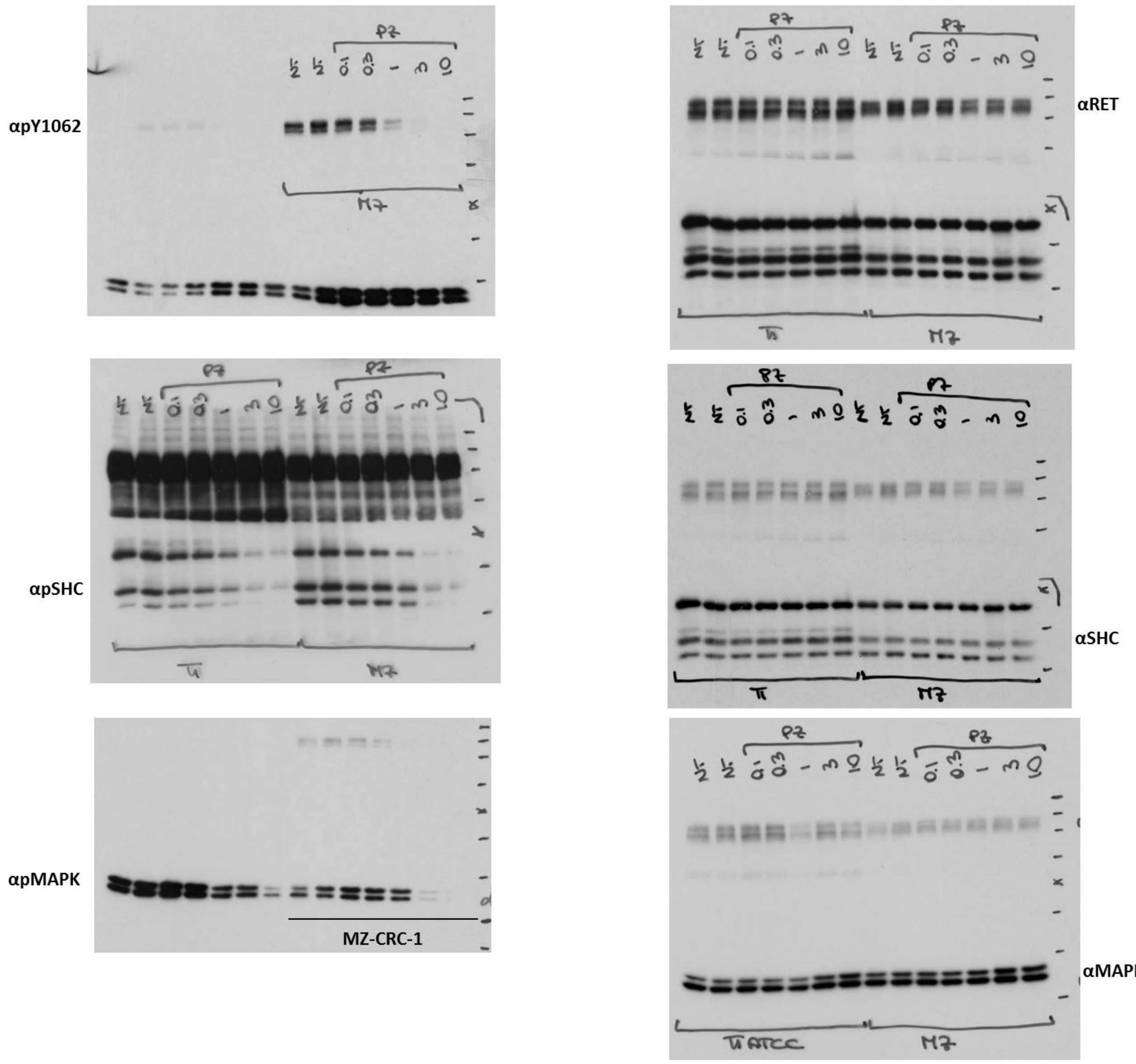

Figure 2 A
TPC-1

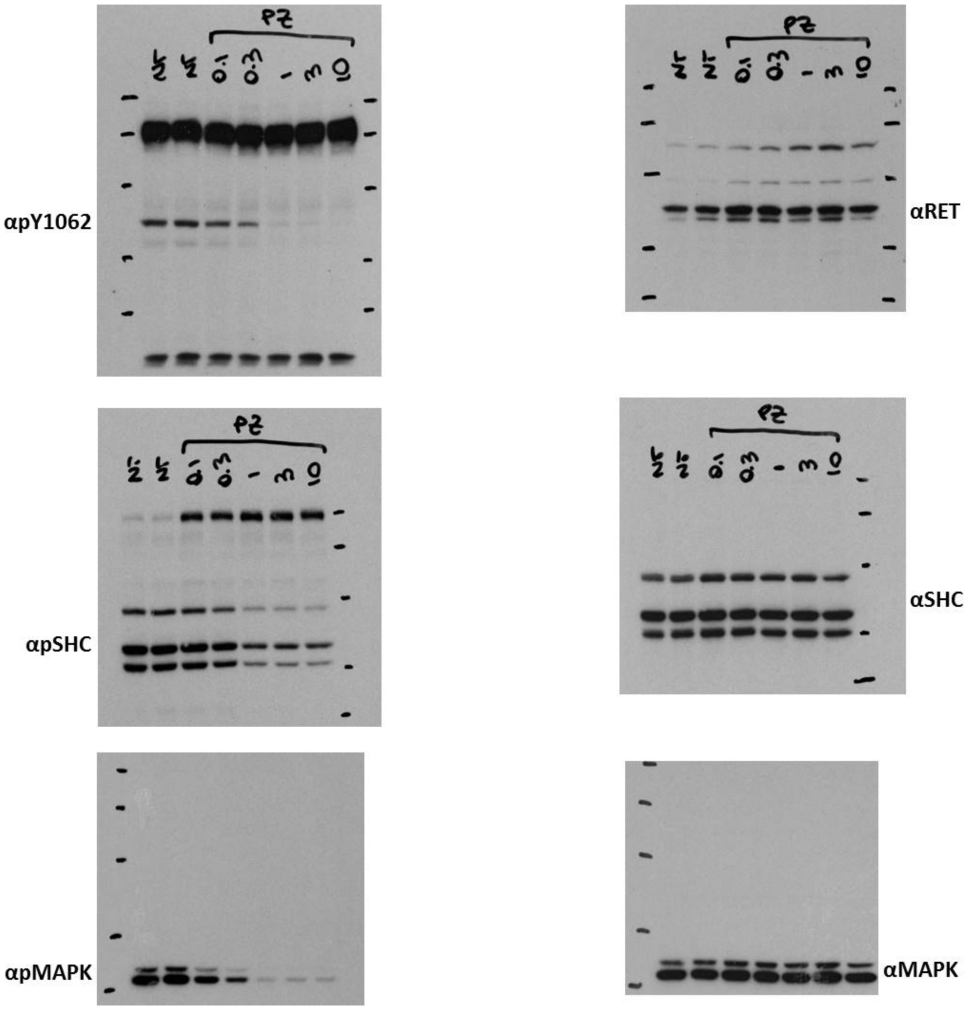

Figure 2 A
TT

αpY1062

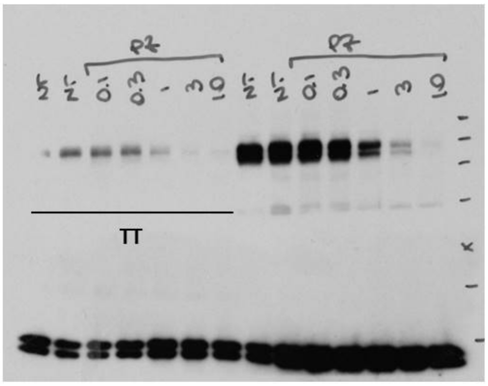

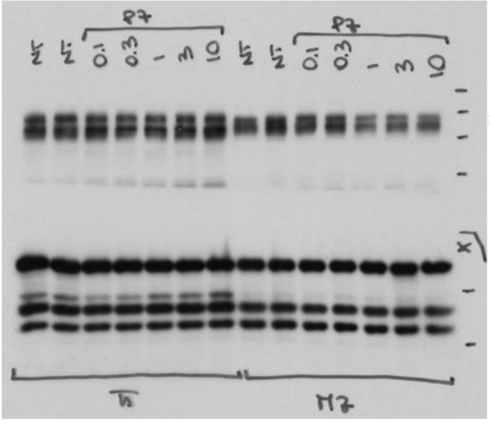

αRET

αpSHC

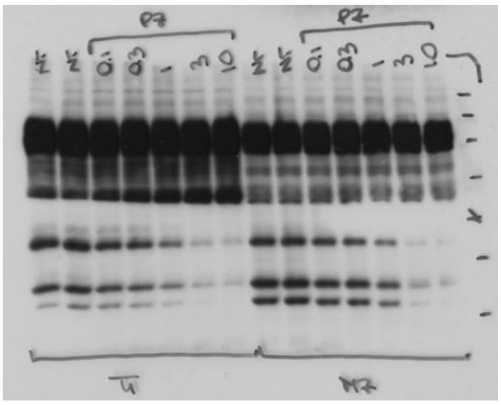

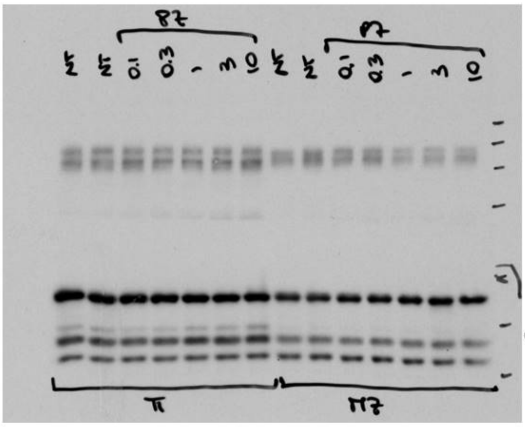

αSHC

αpMAPK

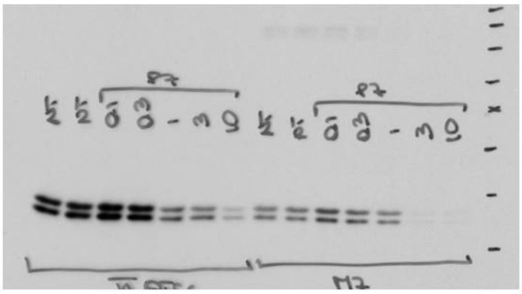

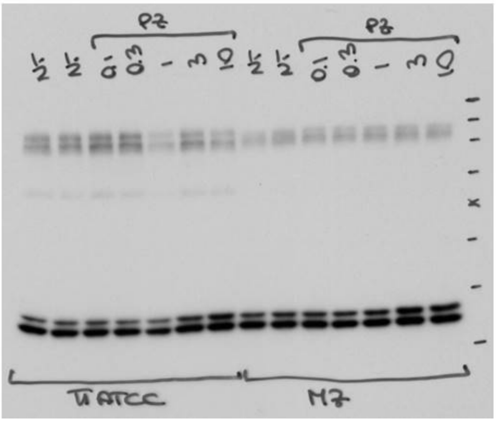

αMAPK

Figure 2 B
Lc-2/ad

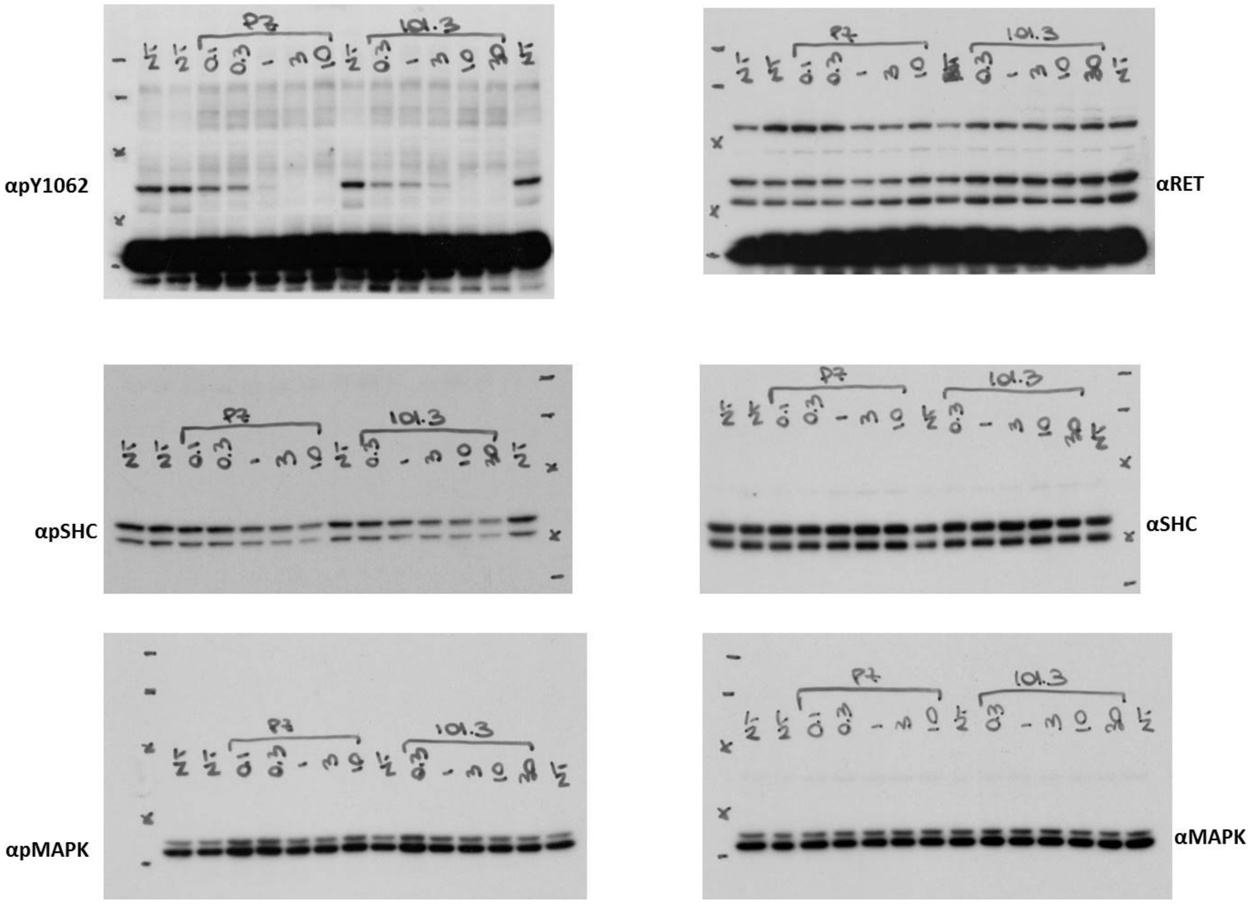

Figure 2 C
KM12

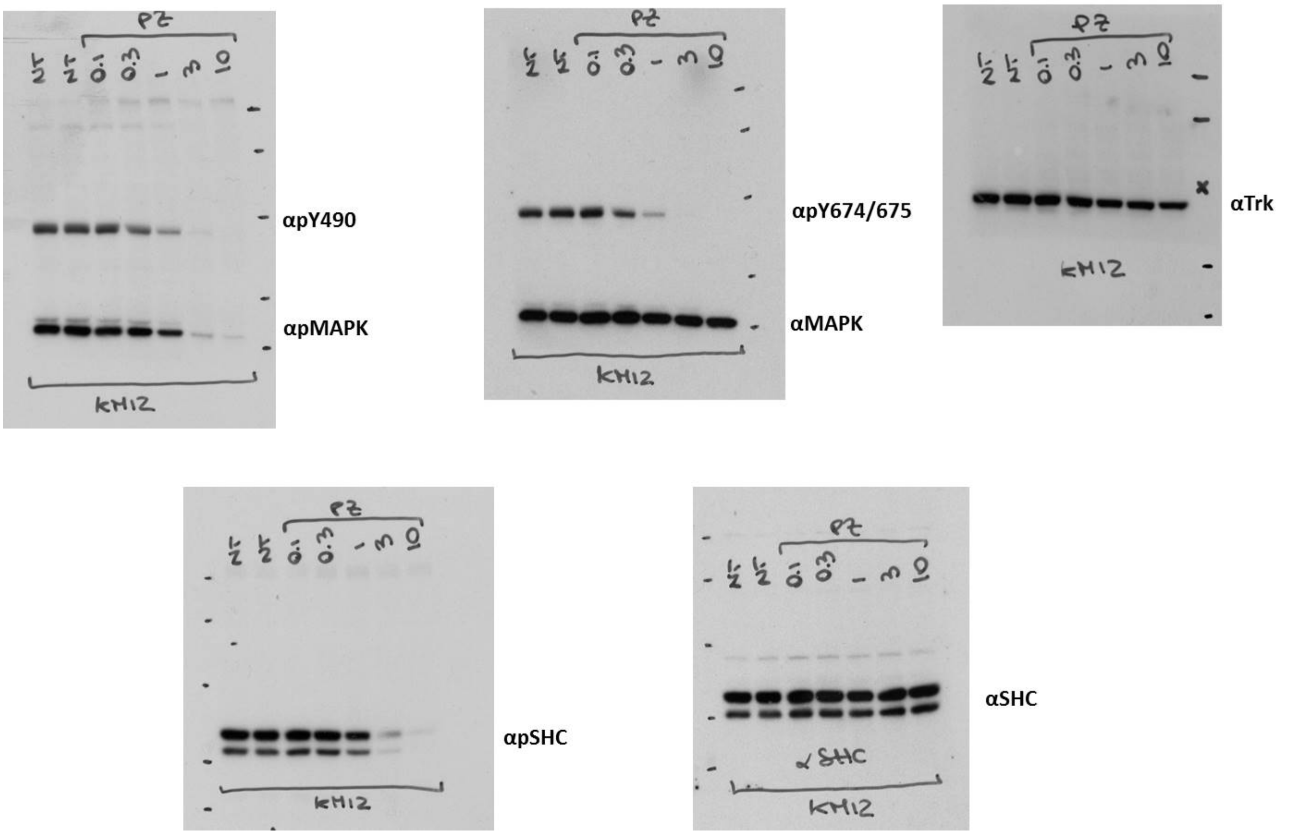

Figure 6 A

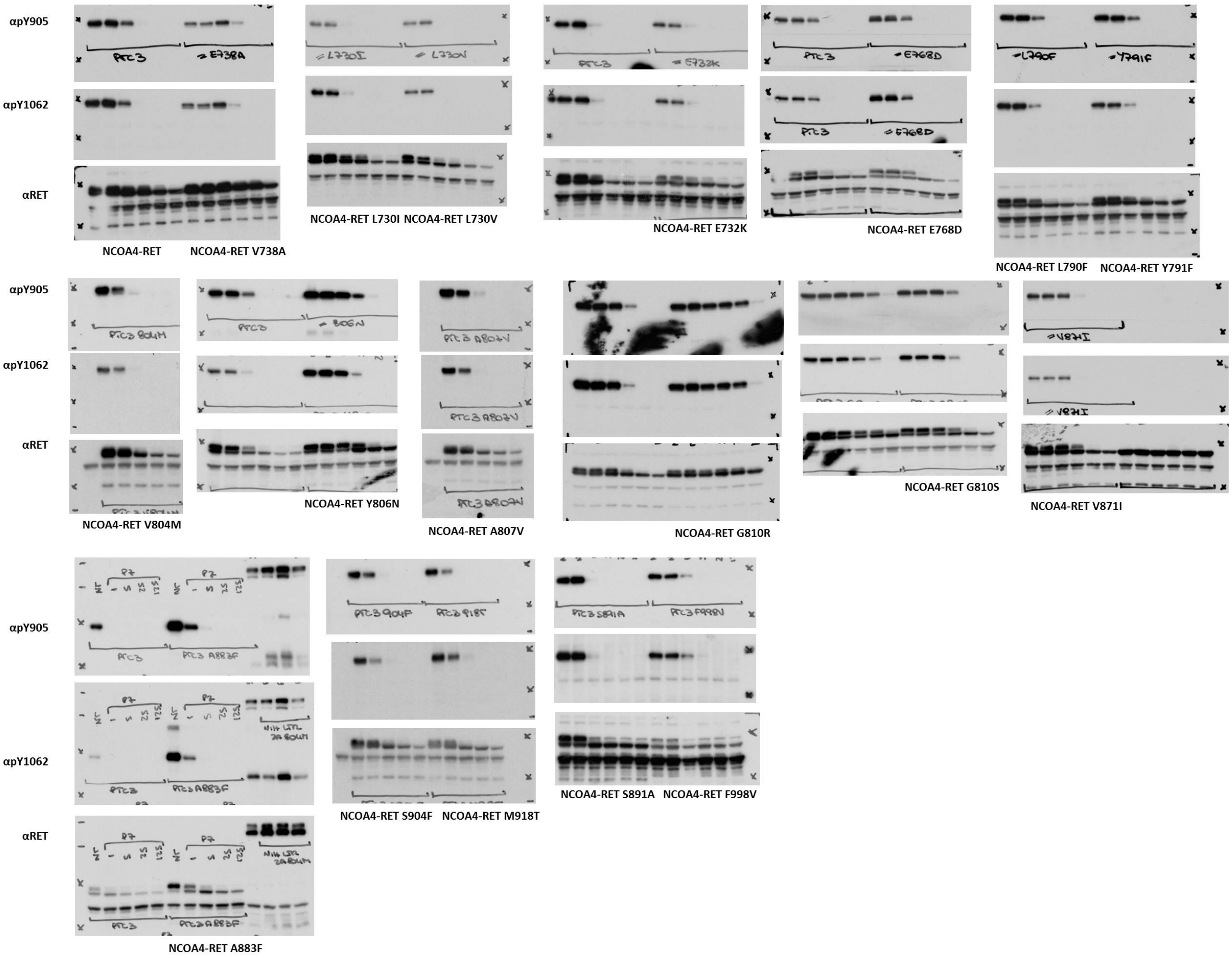
αpY905
αpY1062
αRET
NCOA4-RET
NCOA4-RET V738A
NCOA4-RET L730I
NCOA4-RET L730V
NCOA4-RET E732K
NCOA4-RET E768D
NCOA4-RET L790F
NCOA4-RET Y791F
NCOA4-RET V804M
NCOA4-RET Y806N
NCOA4-RET A807V
NCOA4-RET G810R
NCOA4-RET G810S
NCOA4-RET V871I
NCOA4-RET S904F
NCOA4-RET M918T
NCOA4-RET S891A
NCOA4-RET F998V
NCOA4-RET A883F

Figure 6 B

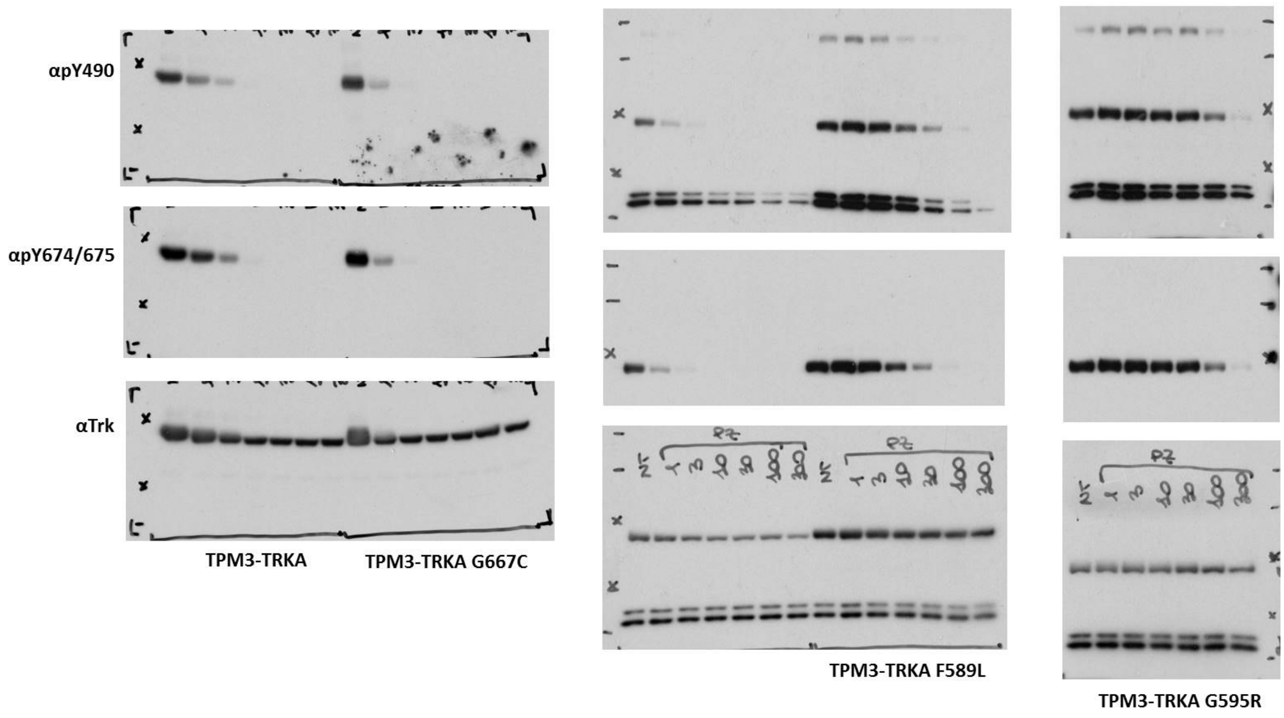

Figure S1

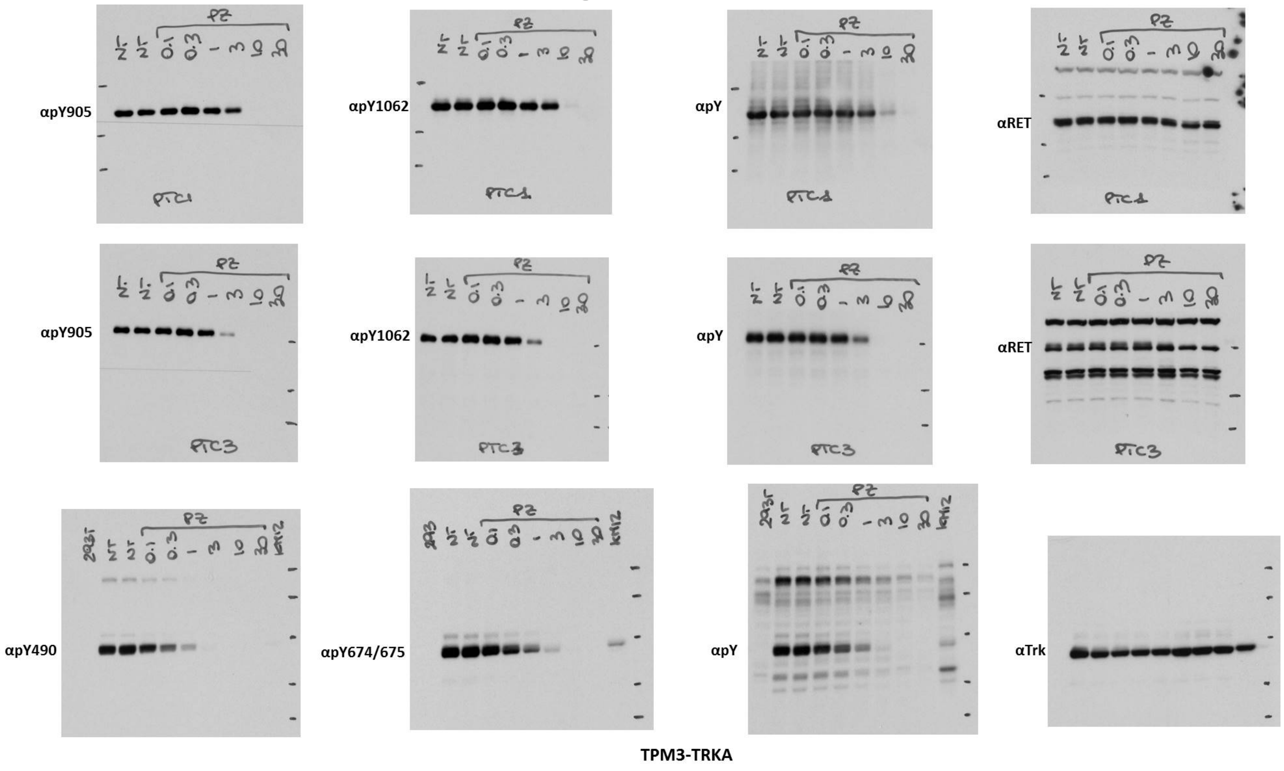

Figure S3

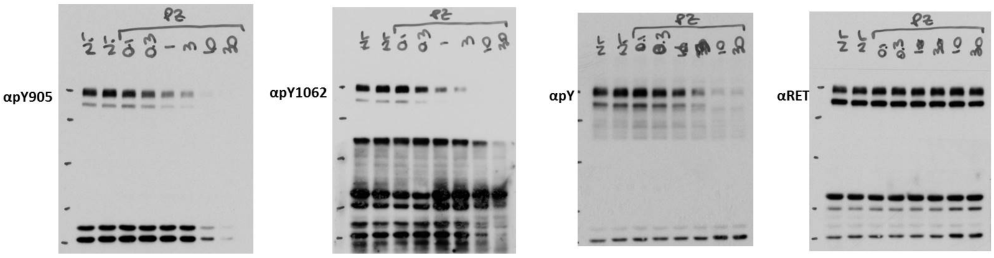

Ba/F3 RET/C634R

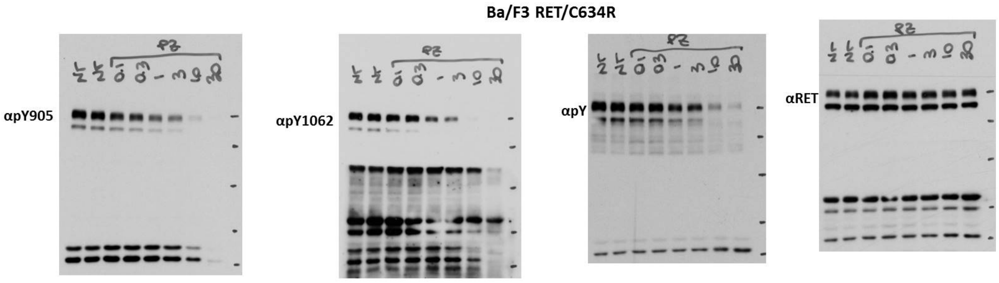

Ba/F3 RET/M918T

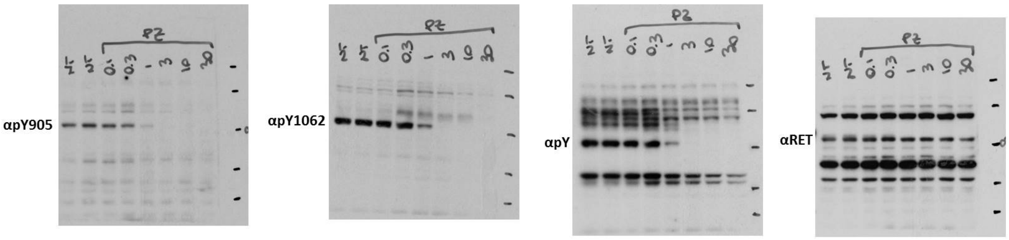

Ba/F3 NCOA4-RET

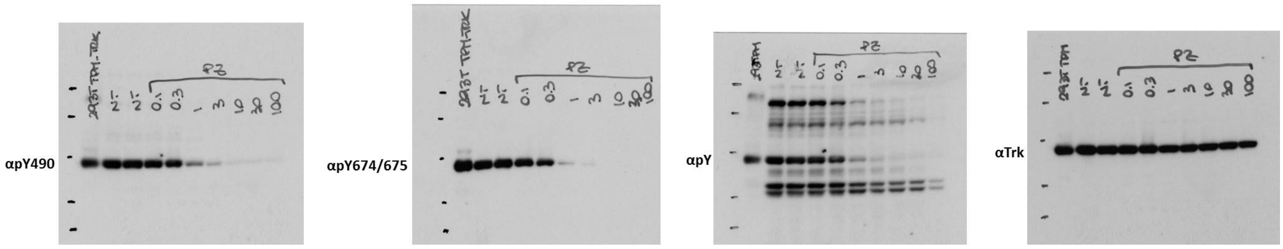

Ba/F3 TPM3-TRKA

Figure S4 A

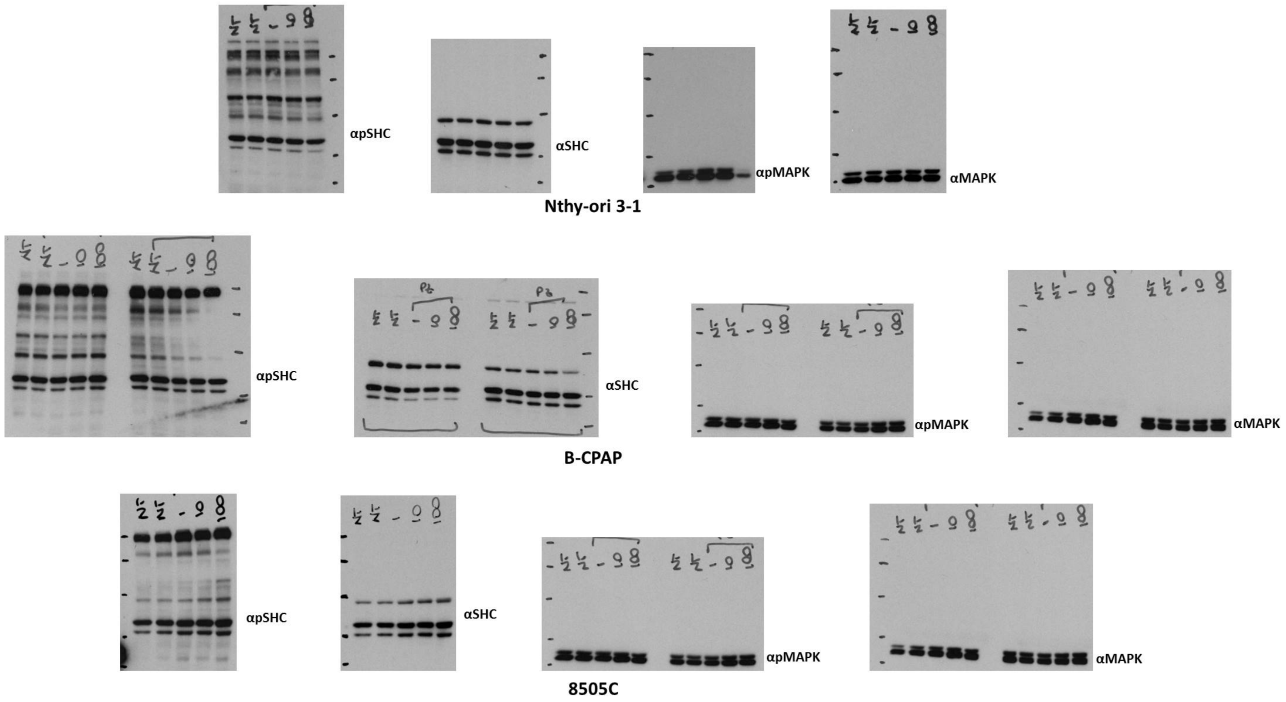
αpSHC
αSHC
αpMAPK
αMAPK
Nthy-ori 3-1
αpSHC
αSHC
αpMAPK
αMAPK
B-CPAP
αpSHC
αSHC
αpMAPK
αMAPK
8505C

Figure S4 B

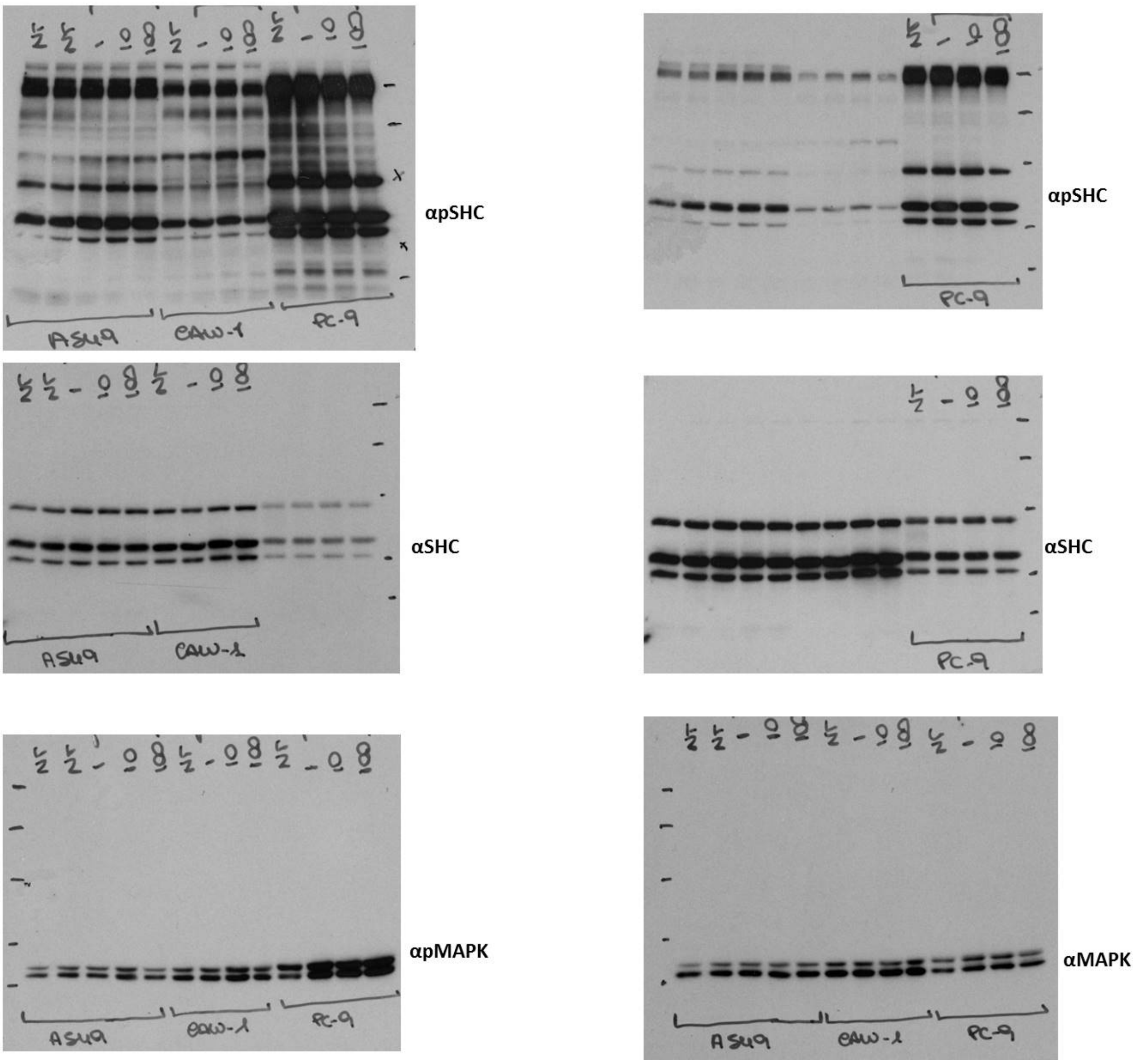

Figure S4 C

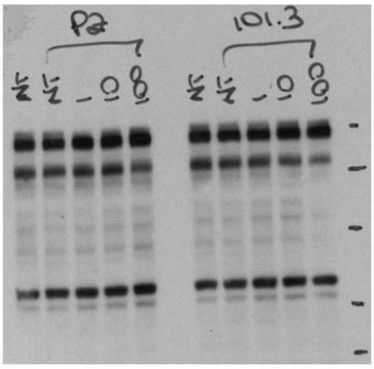

αpSHC

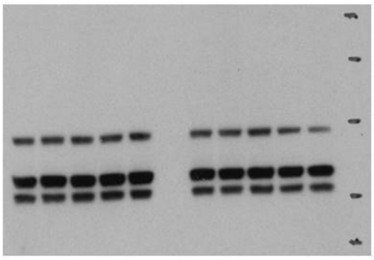

αSHC

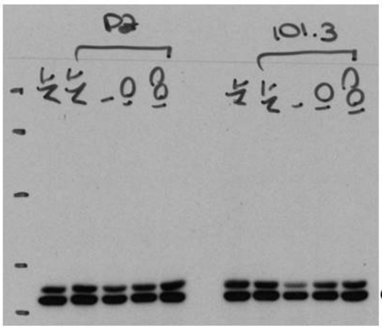

αpMAPK

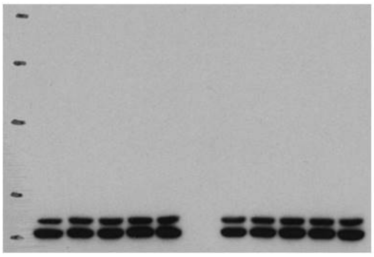

αMAPK

Figure S7
KM12

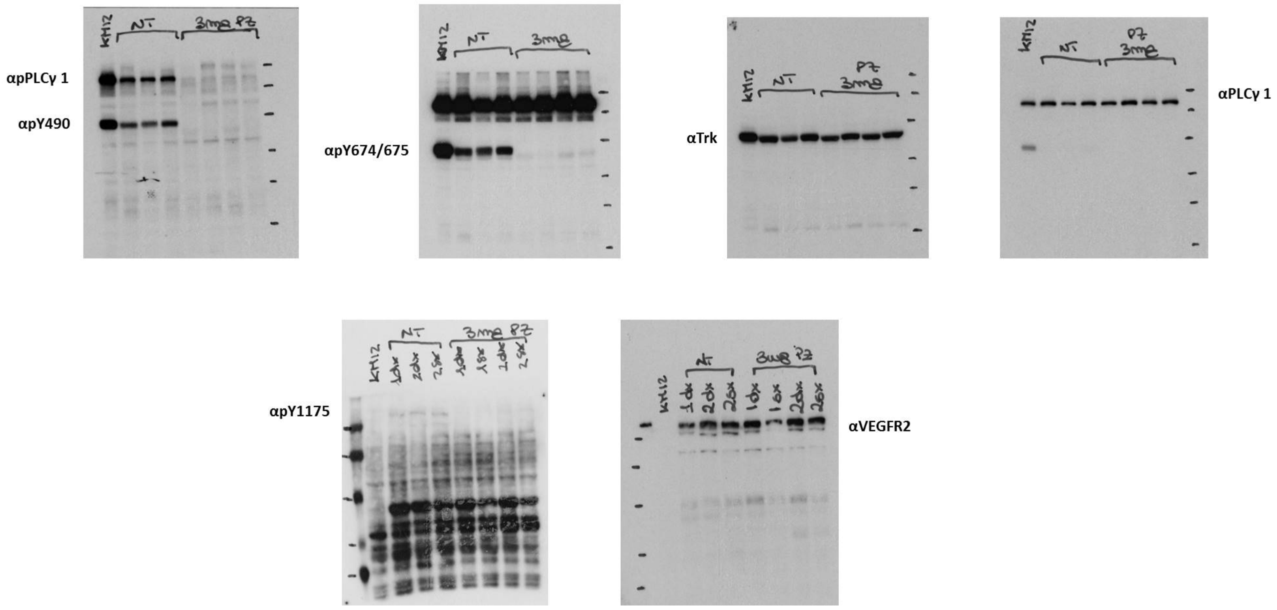

Figure S7
MZ-CRC-1

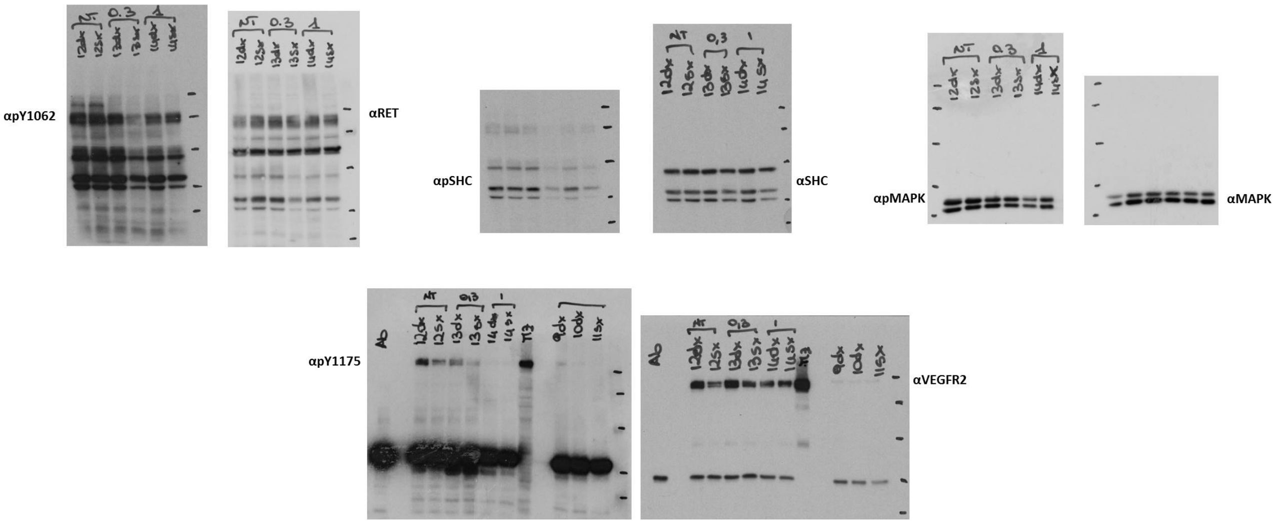

Figure S7
TT

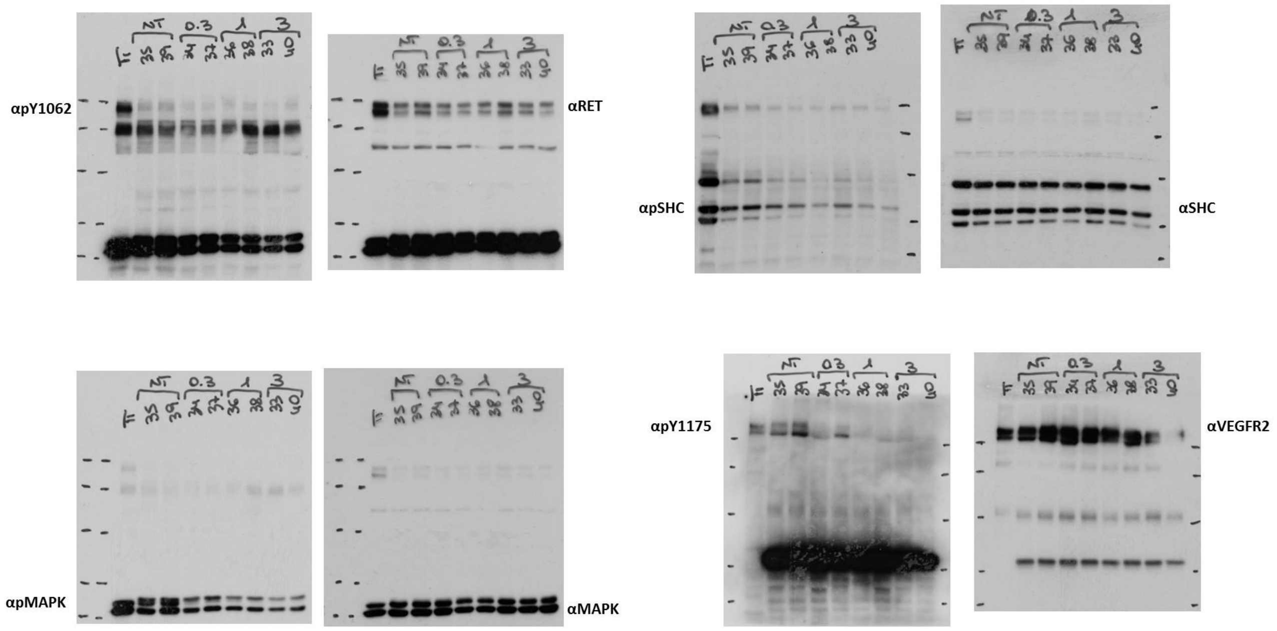

Figure S8
8505C

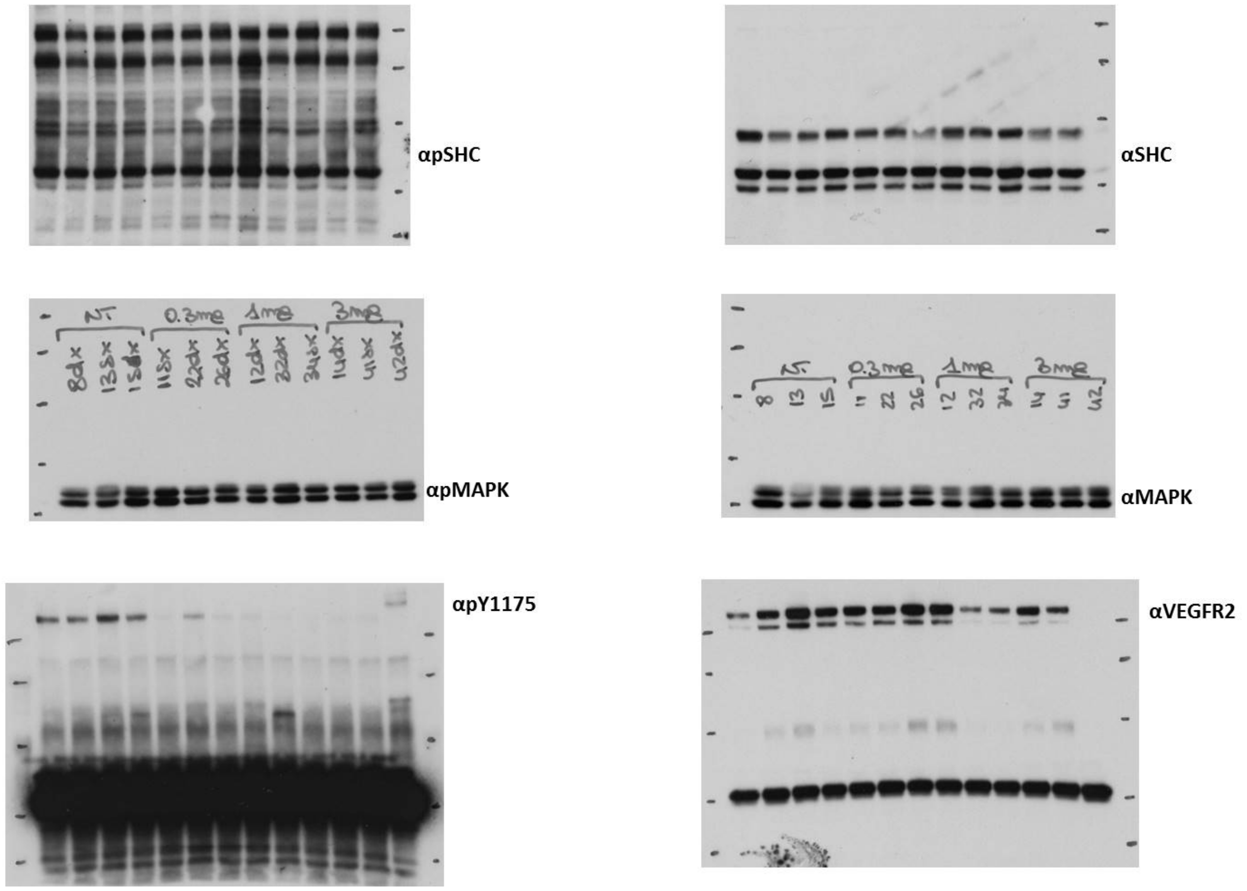

Figure S8
HCT-116

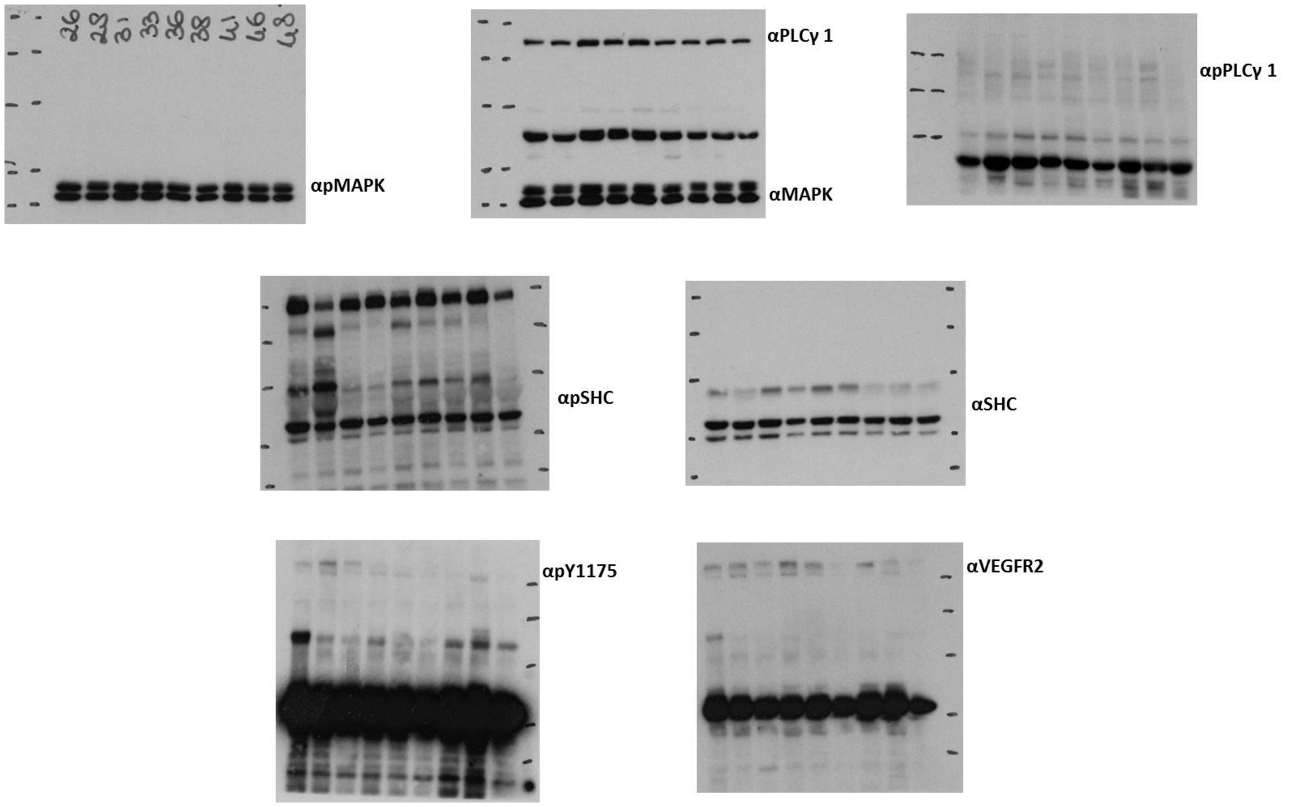

Figure S9

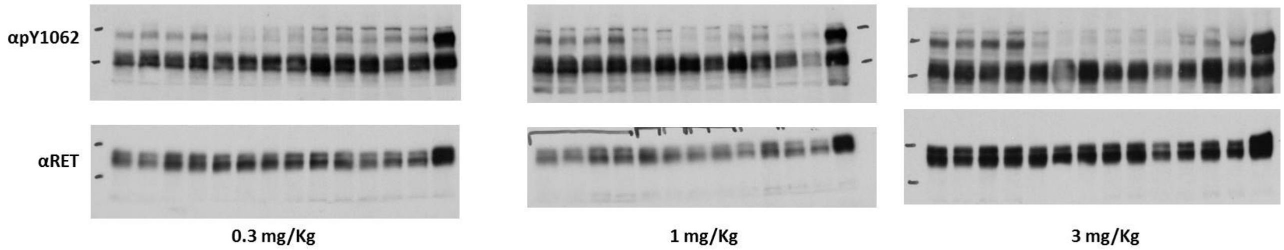

Figure S10

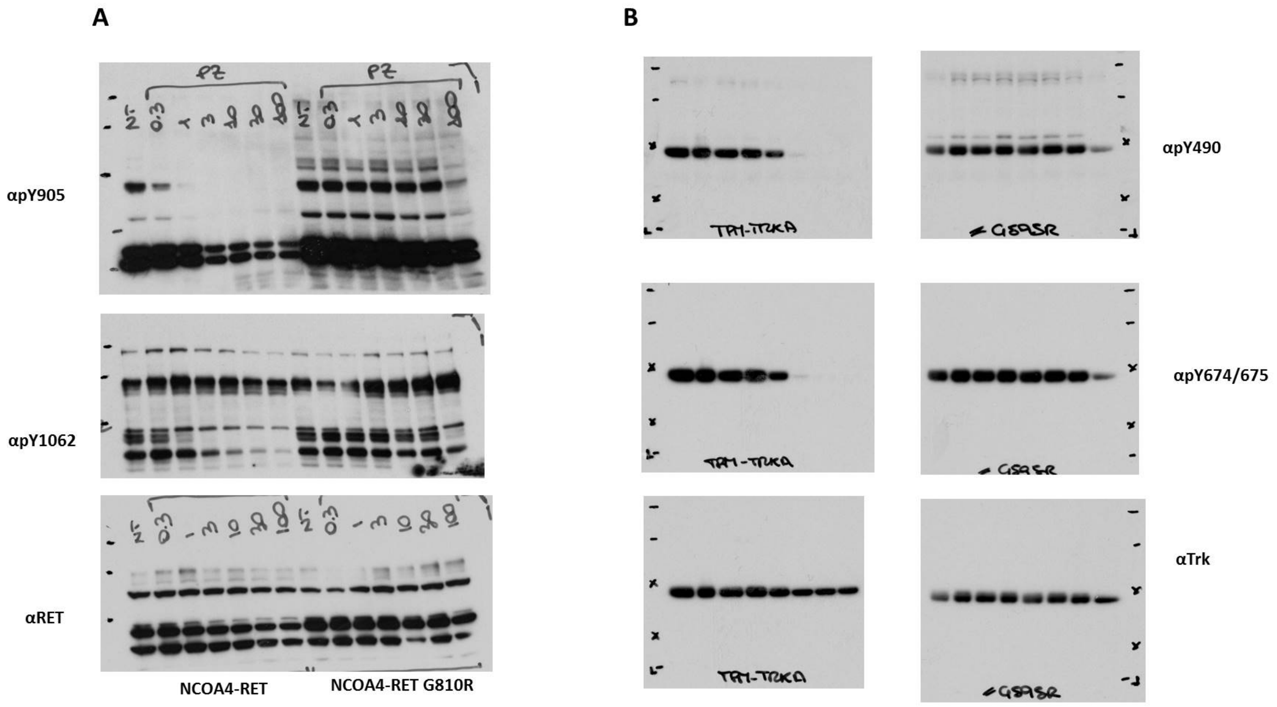

Figure S11

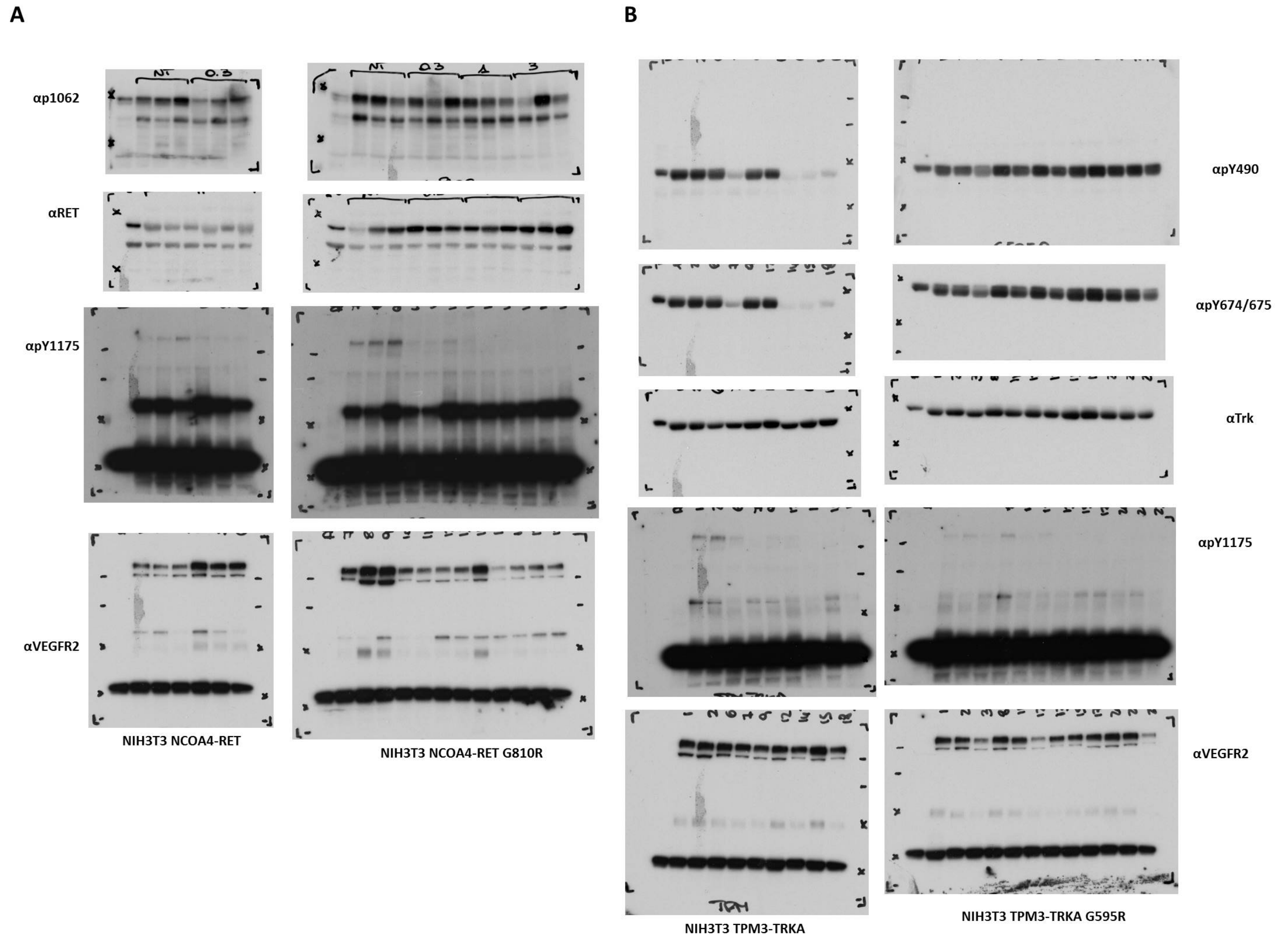

Figure S12

A

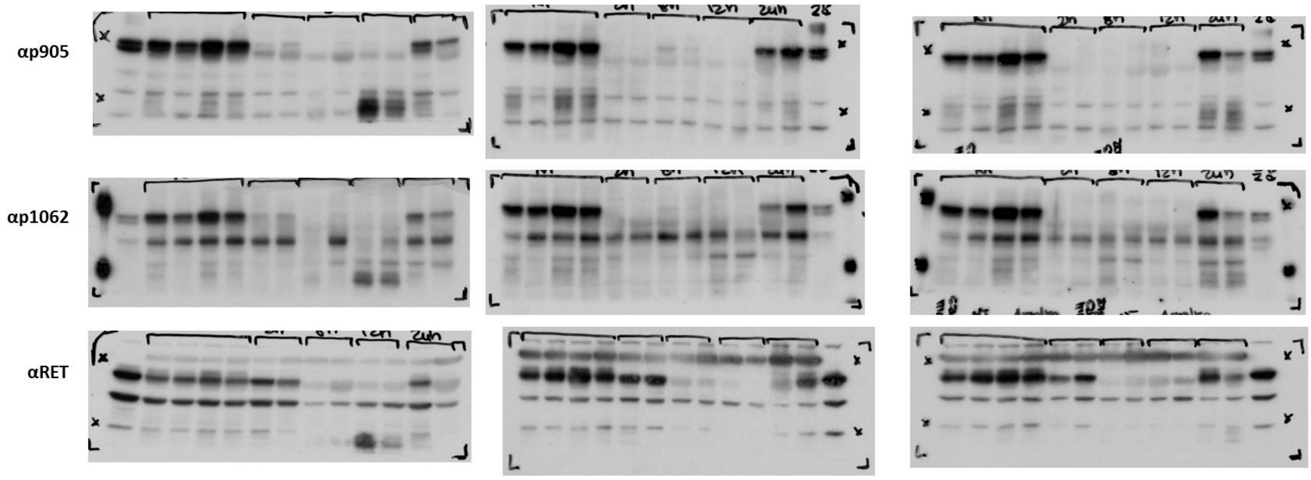

NIH3T3 NCOA4-RET

B

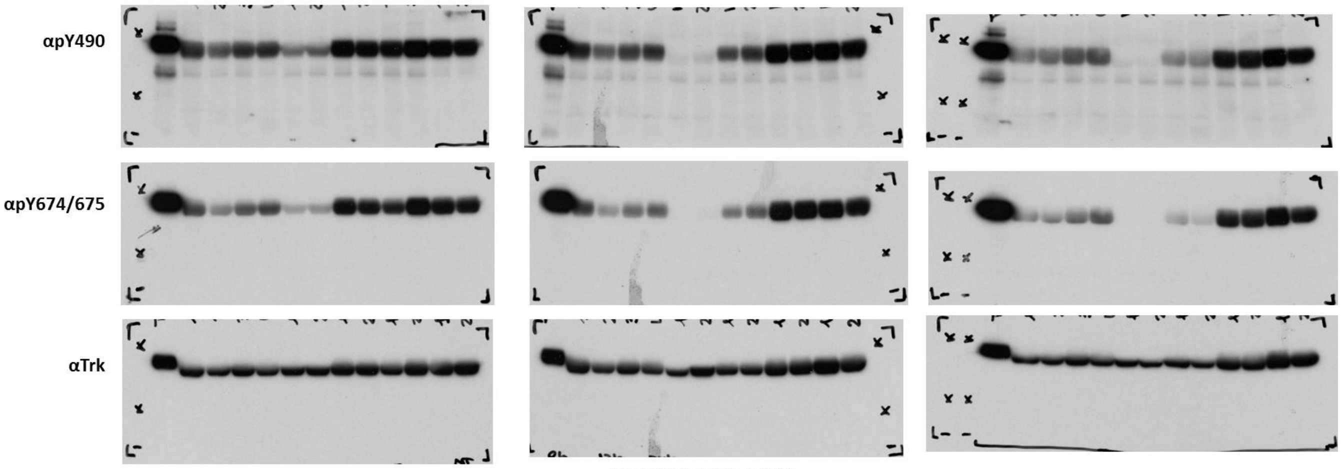

NIH3T3 TPM3-TRKA
